# Three-dimensional in vitro modeling of malignant bone disease recapitulates experimentally accessible mechanisms of osteoinhibition

**DOI:** 10.1038/s41419-018-1203-8

**Published:** 2018-11-26

**Authors:** Eoin P. McNeill, Robert W. Reese, Abishek Tondon, Bret H. Clough, Simin Pan, Jeremiah Froese, Daniel Palmer, Ulf Krause, David M. Loeb, Roland Kaunas, Carl A. Gregory

**Affiliations:** 1 Department of Molecular and Cellular Medicine, Institute for Regenerative Medicine, Texas A&M Health Science Center, College Station, TX 77845 USA; 2 Department of Biomedical Engineering, Texas A&M University, College Station, TX 77843 USA; 3 Department of Medical Physiology, Texas A&M Health Science Center, Temple, TX 76501 USA; 4 Institute for Transfusion Medicine and Transplant Immunology, University Hospital Muenster, Muenster, Germany; 5 Departments of Pediatrics and Developmental and Molecular Biology, Children’s Hospital at Montefiore, Albert Einstein College of Medicine, 3411 Wayne Avenue, Bronx, NY 10467 USA

## Abstract

Malignant bone disease (MBD) occurs when tumors establish in bone, causing catastrophic tissue damage as a result of accelerated bone destruction and inhibition of repair. The resultant so-called osteolytic lesions (OL) take the form of tumor-filled cavities in bone that cause pain, fractures, and associated morbidity. Furthermore, the OL microenvironment can support survival of tumor cells and resistance to chemotherapy. Therefore, a deeper understanding of OL formation and MBD progression is imperative for the development of future therapeutic strategies. Herein, we describe a novel in vitro platform to study bone–tumor interactions based on three-dimensional co-culture of osteogenically enhanced human mesenchymal stem cells (OEhMSCs) in a rotating wall vessel bioreactor (RWV) while attached to micro-carrier beads coated with extracellular matrix (ECM) composed of factors found in anabolic bone tissue. Osteoinhibition was recapitulated in this model by co-culturing the OEhMSCs with a bone–tumor cell line (MOSJ-Dkk1) that secretes the canonical Wnt (cWnt) inhibitor Dkk-1, a tumor-borne osteoinhibitory factor widely associated with several forms of MBD, or intact tumor fragments from Dkk-1 positive patient-derived xenografts (PDX). Using the model, we observed that depending on the conditions of growth, tumor cells can biochemically inhibit osteogenesis by disrupting cWnt activity in OEhMSCs, while simultaneously co-engrafting with OEhMSCs, displacing them from the niche, perturbing their activity, and promoting cell death. In the absence of detectable co-engraftment with OEhMSCs, Dkk-1 positive PDX fragments had the capacity to enhance OEhMSC proliferation while inhibiting their osteogenic differentiation. The model described has the capacity to provide new and quantifiable insights into the multiple pathological mechanisms of MBD that are not readily measured using monolayer culture or animal models.

## Introduction

Approximately 40% of newly diagnosed cancers per year in the US will involve bone, disrupting bone turnover and causing catastrophic damage in the form of osteolytic lesions (OLs)^1^. OLs cause serious fractures and untenable pain, but more importantly, they provide a niche for tumor propagation, reducing the probability of survival^2^. OLs ultimately become persistent hotspots for drug-resistant cell selection and refractory disease^3–7^. It is known that lytic MBD tumors secrete Wnt inhibitors (WI) that inhibit canonical Wnt (cWnt) signalling, a pathway that drives the differentiation of bone marrow mesenchymal stem cells (MSCs) into osteoblasts. Several members of the WI family are involved in OL formation, but Dickkopf-1 (Dkk-1) is the most common, associated with myeloma, osteosarcoma (OS), and breast/prostate cancer metastases^8–10^. While priority is given to reducing tumor load and preventing relapse, promoting repair of OLs is important given that OLs provide an ideal environment for recurrence^2,11^.

Presently, tools to study bone–tumor interactions are limited to tissue culture and animal models. Tissue culture techniques are generally limited to monolayer growth and this does not mimic the three-dimensional (3D) characteristics of tumors and host tissues. Monolayer culture also frequently overestimates responses to experimental drugs^12–14^. An experimentally accessible 3D cell culture system that mimics the bone-tumor microenvironment without the practical and ethical complexities of animal modeling could provide a much-needed alternative to study bone–tumor interactions, especially for rapid study of patient-derived tumor explants^13,14^.

Rotating wall vessel (RWV) bioreactors culture cells under conditions of free fall^15,16^ and are excellent tools for the 3D growth of tissue-like structures due to their superior fluid and gas exchange characteristics and reduced shear exposure as compared to other 3D culture systems^17–19^. Although simulated microgravity has been shown to suppress osteogenesis when compared with monolayer culture on plastic surfaces^20,21^, other studies have shown that 3D culture in RWV can support osteogenesis through the provision of surface topologies and gas/nutrient exchange that better mimic conditions experienced by cells in vivo^22–24^. Herein, we introduce the unexplored concept of co-culturing osteogenically enhanced human MSCs (OEhMSCs) with OS cells and OS-patient-derived xenografts (PDX) using the RWV system. To validate the 3D co-culture system, we employed a clinically relevant and well-studied phenomenon where cWnt inhibitor Dkk-1 secreted by bone–tumor cells inhibits the differentiation of osteoprogenitors^9^. We demonstrate that osteoinhibitory MBD can be recapitulated in the co-culture system using cell lines, PDX fragments, human MSCs and custom attachment surfaces. The described strategy introduces the means to provide new and quantifiable insights into the multiple mechanisms of MBD that are not readily measured using monolayer culture or animal models.

## Materials and methods

### Tissue culture

Human bone marrow-derived mesenchymal stem cells (hMSCs) were acquired from the Texas A & M Health Science Center Institute for Regenerative Medicine Mesenchymal Stem Cell (MSC) distribution facility in accordance with institutionally approved protocols. Culture of the cells was carried out as previously described^25,26^. Briefly, hMSCs were cultured in complete culture medium (CCM) which consisted of alpha-minimal-essential-medium (α-MEM) (Life Technologies, Carlsbad, CA), 20% (v/v) fetal bovine serum (FBS, Atlanta Biologicals, Norcross, GA), 2mM l-glutamine, 100 units per mL penicillin, and 100 µg mL^−1^ streptomycin (Life Technologies). Medium was changed every 2 days. For expansion and storage, hMSCs were recovered using trypsin/ethylene diamine tetra-acetic acid (EDTA) (Life Technologies) when density had reached about 70% or 7,000–10,000 cells per cm^2^. After detachment, hMSCs were either reseeded at 100 cells per cm^2^ or cryopreserved in α-MEM supplemented with 30% (v/v) FBS (Atlanta biologicals) and 5% (v/v) DMSO (Hybrimax, Sigma-Aldrich, St Louis, MO) in the vapor phase of liquid nitrogen. Green fluorescent protein (GFP)-positive hMSCs at passage 5 were used for the experiments. Phase contrast and fluorescence microscopy of live cell cultures was carried out using an inverted microscope (Nikon Eclipse TE200) fitted with a Nikon DXM1200F digital camera.

### Labeling of MOSJ cells and hMSCs

MOSJ cells were modified with constructs encoding Dkk1 and dsRed2 fluorescent protein as previously described^26^. Control MOSJ cells were also generated that were dsRed2 labeled, but harbored only the vector backbone (MOSJ-pLenti). The hMSCs were labeled with enhanced green fluorescent protein (eGFP) by lentiviral transduction with the construct that drives GFP cDNA under the constitutive chicken actin promoter pWPT-GFP (Trono Laboratory). A MoFlo XDP fluorescence activated cell sorter (FACS) (Beckman Coulter, Indianapolis, IN) was used to generated a cell bank with >99% purity.

### Live/dead staining of cells on micro-carrier beads

A Live/Dead Cell Imaging Kit (Life Technologies) was employed to assess the viability of both the hMSCs and MOSJ cells on the beads. The assay was carried out according to the manufacturer’s instructions with the exception of the use of Hoescht 33342 dye (Sigma) to visualize dead MOSJ cells as it was not possible to distinguish between the propidium iodide stain and the dsRed2 already expressed by the cells. Live staining was carried out on hMSCs not expressing GFP to allow visualization of the calcein AM dye that indicates live cells.

### Immunophenotyping

hMSCs were recovered by briefly incubating with trypsin/EDTA (Life Technologies). A single-cell suspension was then incubated on ice for 30 min in PBS containing 2% (v/v) FBS with fluorophore-tagged antibodies or their isotype controls (Becton Dickinson, Franklin Lakes, NJ or Beckman Coulter). Antibodies against CD11b (clone BEAR1), CD14 (RMO52), CD19 (J3-119), CD34 (581), CD45 (J.33), CD73 (AD2), CD79a (HM47), CD90 (Thy-1/310), CD105 (IG2), and HLA-DP, DQ, DR (Tu39) were used. A Cytomics FC500 flow cytometer (Beckman Coulter) was used to analyze the cells (minimum of 20,000) and the data were processed using the manufacturer’s software (CXP).

### Osteogenic differentiation and Alizarin Red S staining

Osteogenic differentiation and staining was carried out as previously described^27^. All reagents were acquired from Sigma Aldrich unless otherwise stated. Confluent monolayers of hMSCs were incubated in CCM supplemented with 20% (v/v) FBS (Atlanta Biologicals) containing 100 nM dexamethasone, 50 µg mL^−1^ ascorbic acid, and 5 mM β-glycerol phosphate for 3 weeks with fresh media exchanged every two days to stimulate osteogenic differentiation with mineralization. The monolayers were then washed twice with PBS and fixed with 10% (v/v) neutral buffered formalin (Sigma) for 15 min at room temperature. The fixed monolayers were stained with 40 mM Alizarin Red S pH 4.0 (Sigma) for 30 min. They were then washed with distilled water four times before micrographs with obtained using an inverted microscope (Nikon Eclipse, TE200) fitted with a Nikon DXM1200F digital camera.

### Adipogenic differentiation and Oil Red O staining

Confluent monolayers of hMSCs were incubated in CCM containing 0.5 µM dexamethasone, 50 nM isobutylmethylxanthine, and 500 nM indomethacin (Sigma) for 3 weeks with fresh media exchanged every two days to stimulate adipogenic differentiation. The monolayers were then washed twice with PBS and fixed with 10% (v/v) neutral buffered formalin for 15 min at room temperature. The fixed monolayers were then stained with 0.5% (w/v) Oil Red O solution (Sigma) in 30% (v/v) isopropanol in PBS for 20 min. Excess stain was then washed from the monolayers with PBS and micrographs were taken using an inverted microscope (Nikon Eclipse, TE200) fitted with a Nikon DXM1200F digital camera.

### Chondrogenic differentiation and processing

250,000 hMSC were pelleted by centrifugation for 10 min at 500 × *g* and incubated in high-glucose Dulbecco’s minimal-essential-media (Life Technologies) containing 1 µM dexamethasone, 50 µg mL^−1^ ascorbate-2-phosphate, 40 µg mL^−1^ proline, 100 µg mL^−1^ pyruvate, and 2x Insulin Transferrin Selenium-Plus Premix (Sigma) for 3 weeks with fresh media exchanged every 3 days to stimulate chondrogenic differentiation. The pellets were then washed in PBS and fixed in 4% (v/v) paraformaldehyde for 15 min at room temperature. The fixed pellets were then embedded in paraffin, sectioned, and stained with toluidine-borate solution to visualize sulphated proteoglycan deposition.

### Extracellular matrix (ECM) production

ECM was produced as previously described^28^. Reagents were sourced from Sigma unless otherwise stated. Unlabeled hMSCs were grown as monolayers to 70–80% confluency (~15,000 cells per cm^2^) in CCM before exposure to osteogenic enhancement media (OEM) consisting of CCM supplemented with 50 µg mL^−1^ ascorbic acid, 5 mM β-glycerol phosphate and 10 µM GW9662. The media was changed every 2 days for 10 days. The monolayers were then washed with PBS and placed in a −80 °C freezer for 15 h to disrupt the cell membranes. The monolayers were thawed, washed with PBS and scraped from the tissue culture plate. The material from about 150 cm^2^ of monolayer was recovered by centrifugation at 1000 × *g* for 15 min and suspended in 10 mL of lysis buffer consisting of PBS containing 0.1% (v/v) Triton X100, 1 mM MgCl_2_, 10 µg mL^−1^ DNAse I. Ten to 30 mL of lysis mixture was placed in a 50 mL centrifuge tube and incubated horizontally at 37 °C with orbital mixing at 60 rpm for 4 h. Trypsin was then added to the lysis buffer for a concentration of 0.1% (v/v) and the reaction was allowed to continue for a further 16 h. The resultant ECM was then recovered by centrifugation, washed in excess dH_2_O twice followed by one wash with chloroform. Finally, the ECM was washed in acetone and allowed to air dry. For solubilization, ECM pellets were suspended and dispersed in 0.1 M ice cold acetic acid (0.6% v/v) with least 10 mg ECM per mL of acetic acid. Suspensions were sonicated in a cold water bath sonicator (Bransonic, Danbury, CT) with 6 × 5 s bursts over 60 s initially, then at 15 h, and finally at 30 h. During the 30 h period, the solutions were stored at 4 °C with rapid stirring. On average, solutions of 3–5 mg mL^−1^ could be attained.

### Preparation of ECM-coated beads

Reagents were sourced from Fisher Scientific unless otherwise stated. Enhanced Attachment Microcarriers (Corning, Corning, NY) were used for ECM attachment with a size range of 125–212 μm, density of 1.026 g per cm^3^, and 360 cm^2^ growth area per gram. Prior to use, the beads were washed thoroughly with sterile deionized water and suspended to a final concentration of 200 mg mL^−1^. Ten mg of *N*-(3-Dimethylaminopropyl)-*N*′-ethylcarbodiimide hydrochloride (EDAC) and 10 mg of *N*-Hydroxysuccinimide (NHS) were dissolved in 10 mL of 0.1 M 2-(*N*-morpholino)ethanesulfonic acid (MES) buffer containing 0.9% (w/v) sodium chloride (pH 4.7) and filter sterilized. The EDAC/NHS/MES solution was added to the beads and the reaction was allowed to proceed for 15 min with shaking at room temperature. The beads were then recovered by centrifugation, washed with sterile PBS, and then reconstituted in PBS containing ECM at a concentration of 1 mg mL^−1^. ECM was allowed to react with the beads for 2 h on a shaker, in the dark at room temperature. The bead solution was centrifuged, washed with PBS, and finally reconstituted in sterile PBS for storage at 4 °C until use. Equivalent collagen I-coated control beads were also acquired from Corning.

### Preparation of coated tissue culture plates

A solution of 25 µg mL^−1^ collagen I (from rat tail, Sigma Aldrich, St Louis, MO) or ECM from OEhMSCs was prepared in dH_2_O. One half-mL of the solution was dispensed into each well of a 12-well plate. The plates were left at 4 °C for at least 24 h. The plates were washed with PBS prior to cell seeding.

### Immunostaining

Immunostaining was performed as described previously^28^. A mouse monoclonal antibody directed against the human collagen Iα1 chain (Abcam, Cambridge, UK, ab90395) was used at 1:500 dilution followed by detection by a goat anti-mouse antibody conjugated to Alexafluor-488 (Thermo Fisher). Images were captured using an inverted microscope (Nikon Eclipse TE200) fitted with a Nikon DXM1200F digital camera.

### Electron microscopy

Scanning electron microscopy (SEM) of purified ECM preparations was outsourced to RealView Analytical Laboratory (Roslindale, MA). Samples were washed through an escalating series of ethanol concentrations (50–100% (v/v)) and air dried. A thin layer of carbon (~10 nm) was then coated onto samples by a Denton Thermo vacuum evaporator and the samples were observed under an FEI/Philips XL30 FEG scanning electron microscope. Coated beads were fixed in 4% (v/v) glutaraldehyde and prepared for SEM by ethanol dehydration and coating with gold-palladium alloy as previously described^29^. Beads were visualized using a FEI Quanta 600 scanning electron microscope.

### Enzyme-linked immunosorbent assays (ELISA) for osteoprotegerin (OPG) and Dkk-1

A human OPG antibody duo-kit was obtained from (R&D Systems, Minneapolis, MN) and the assay was carried out according to the manufacturer’s instructions on media diluted 1:5 with PBS containing 5% bovine serum albumin and 1% (v/v) Tween 20 (Sigma). A human Dkk-1 duo-kit was obtained from (R&D Systems) and the assay was carried out according to the manufacturer’s instructions on undiluted media conditioned for 2–3 days.

### Co-culture of hMSCs and MOS-J cells

Schematics of the experimental design are presented in Fig. S2A-C. Prior to loading in the RWV bioreactors, the cells were expanded by conventional low-density monolayer cell culture to obtain the required numbers. To load the beads, collagen I- or ECM-coated beads with a combined growth area of 50 cm^2^ and 2 × 10^6^ GFP-labeled hMSCs were incubated in a square, 100 cm^2^ low-adherent polystyrene plate at 37 °C for 2 h in 10 mL CCM with orbital mixing at 30 revolutions per minute. The beads were recovered by centrifugation at 50 × *g* for 30 s then washed twice with PBS to remove unattached cells. The loaded beads were then suspended in 10 mL of CCM and transferred into the RWV culture system. For this purpose, a Synthecon RCCS-8DQ bioreactor (Synthecon, Houston, TX) fitted with 8 disposable 10 mL high aspect ratio vessels (HARVs) was employed (Fig. S2D). Rotation was initially set to 12 rpm and monitored closely so as to ensure free fall and minimize contact with the walls of the vessel. After 48 h of equilibration, the CCM was removed and replaced with OEM so as to generate OEhMSCs throughout the course of the experiment. For co-culture experiments, dsRed-labeled MOSJ cells were loaded onto beads using the same method as the hMSCs but in this case, 400,000 MOSJ cells were loaded onto a total combined growth area of 10 cm^2^. The MOSJ laden beads were added to the HARVs 24 h after the hMSCs, halfway through the equilibration period, 24 h before addition of OEM. Eighty percent of the OEM media was replenished every 2 days. Entire cultures (*n* = 3) were harvested at day 0 (at the time of OEM addition) and at day 4 and 8 post addition of OEM. For this purpose, 5 mL media was cleared by centrifugation and retained for enzyme-linked immunosorbent assay (ELISA). The cell-laden beads were then recovered by centrifugation, washed in PBS and gently dissociated by trituration. Twenty percent of the beads were subjected to alkaline phosphatase enzymatic assay, 75% were cryopreserved in liquid nitrogen for RNA extraction and approximately 5% was visualized by microscopy. Monolayer controls were performed in 50 cm^2^ plates or 12-well plates (Corning) in exactly the same way, but MOSJ cells were added directly and cells were recovered by trypsinization.

### Alkaline phosphatase (ALP) assays

For monolayer culture, ALP assays were performed as previously described^28,30^. Briefly, OEhMSCs were cultured in the presence or absence of MOSJ-Dkk1 or MOSJ-pLenti cells for up to 8 days in 12-well plates. On days 0, 4, or 8 the monolayers (*n* = 4 wells) were washed twice with PBS, then once with ALP reaction buffer (100 mM Tris-HCl, pH 9, 100 mM KCl and 1 mM MgCl_2_). One half-mL ALP buffer was then added, immediately followed by 0.5 mL of p-nitrophenyl phosphate (PNPP, Life Technologies) A FluoStar plate reader (BMG Biotech) was used to record the absorbance at 405 nm every 30 s for 10 min. For normalization of the measurements, the cells were recovered by trypsinization and enumerated by the fluorescence signal generated by the dsRed2 (558/583) or eGFP (488/510) labeled MOSJ cells or hMSCs respectively using the microplate reader. Standard curves for this assay are presented in Fig. S3C, D. For RWV culture, 20% of the beads were washed twice with PBS and once with ALP buffer. One mL of ALP buffer was then added to the sample with 1 mL PNPP. One-hundred µL of the sample was transferred to a microcentrifuge tube containing 100 µL 1 N NaOH stop solution every minute for 10 min. The samples were then centrifuged through polyethersulfone spin filter (0.45 µm) and transferred to a 96-well plate (Corning). A FluoStar plate reader (BMG Biotech) was then used to read the absorbance at 405 nm. The results were normalized using the cell enumeration obtained by qRT-PCR for GAPDH transcription (Fig. S3A,B).

### Transcriptome profiling

Two million GFP-labeled hMSCs were attached to collagen I- or ECM-coated beads with a combined growth area of 50 cm^2^. The loaded beads were then suspended in 10 mL of CCM and transferred into the RWV culture system. After 48 h of equilibration, the CCM was removed and replaced with OEM so as to generate OEhMSCs. The cultures were incubated for 8 days with changes of media every 2 days. Triplicate cultures for collagen-I-coated and ECM-coated beads were performed. After 8 days, the cell-laden beads were recovered by brief centrifugation, washed in PBS and subjected to total RNA purification. Resultant total RNA yields ranged from 4.5 to 13.5 μg at a concentration of 150–450 μg mL^−1^ with OD260/280 ratios ranging between 1.85 and 2.0. Thereafter, sample preparation and data acquisition was performed by the UT Southwestern Genomics and Microarray Core Facility. RNA integrity was confirmed by analysis using an Agilent 2100 bioanalyzer (Agilent Technologies, Santa Clara, CA) and RNA integrity scores were 10 in each case. Biotin-UTP-labeled antisense copy RNA (cRNA) was generated from 200 ng of total RNA using a commercially available kit (Illumina TotalPrep RNA Amplification Kit, Life Technologies, Carlsbad, CA). Labeled cRNA was hybridized to a HumanHT-v4.0 Expression BeadChip (Illumina, San Diego, CA) and visualized with biotin-Cy3 (Amersham, Piscataway, NJ, USA). Chips were read on an Illumina Hiscan scanner and analyzed according to standard manufacturer’s protocols using GenomeStudio version 3 (Illumina). Background correction, quality control, and quantile normalization were performed in accordance with Illumina standard operating procedures. Mean normalized fluorescent intensities and standard deviations were calculated for each transcript using biological triplicates for each condition. Data for a given transcript were excluded if the standard deviations exceeded 0.25 of the mean. Linear fold changes were calculated between type I collagen and ECM coatings using mean intensity values and lists were compiled of those genes that were upregulated by 2-fold or higher. Gene ontology clustering, tissue expression profiling and pathway analysis was performed using the Database for Annotation, Visualization and Integrated Discovery (DAVID) package version 6.8^31,32^.

### Quantitative real time polymerase chain reaction (qRT-PCR)

Total RNA was extracted from cells using a High Pure RNA isolation kit (Roche Diagnostics, Indianapolis, IN). A Superscript III kit (Life Technologies) was used according to the manufacturer’s protocol to synthesize cDNA. The use of a random hexamer/oligo-dT combination was the only deviation from this protocol. For qRT-PCR amplification, 0.5 μg of cDNA was amplified in a 25 μL reaction containing SYBR Green PCR master mix (Fast SYBR Green, Applied Biosystems) on a C1000 thermocycler fitted with a real-time module (CFX96, Biorad). Species-specific primers were used according to the conditions described in Table S1. GAPDH cycle thresholds were used to enumerate cells by plotting 1/logCt by cell number on standard curves (Fig. S2C&D). The relative expression of the osteogenic and Wnt-responsive transcripts were calculated using the 2^−ΔΔCT^ method^33,34^. For heat map generation, the data were log-transformed and z-normalized to the same scale, then plotted using Rstudio (v1.1.435), ggplot (3.0.0), dplyr (0.7.6) and reshape (1.4.3) programs.

### Patient-derived xenograft (PDX) specimens

PDX specimens were provided by the Loeb laboratory. The specimens were recovered from surgically excised pediatric OS specimens in accordance with an institutionally approved human research protocol. The specimens were expanded in immune deficient murine hosts as previously described and were demonstrated to secrete Dkk-1 in vivo^35^. PDX samples were recovered from cryoprotectant, sliced into ~1 cubic mm morsels and cultured in a HARV containing CCM for up to 10 days. Viability was visualized by calcein AM staining using a commercially acquired kit (Live/Dead kit, Thermo Fisher, Waltham, MA) and quantified by fluorescence measurements at 494/517 using a microwell plate reader (Fluostar Optima, BMG Biotech). Glucose and lactate levels on media were quantified using blood analyzers (Bayer, Pittsburgh, MA and Nova Biomedical, Waltham, MA).

### Statistics

GraphPad Prism version 5.00 for windows was used to plot the data and carry out statistical analysis. Normality of distribution and equivalence in variability was calculated by GraphPad software. One-way analysis of variance (ANOVA) was used to analyze multiple tests of means within data sets with Tukey post testing where necessary. *t*-tests were used to compare single means. Experiments were performed twice and in some cases in different laboratories. *N* = 6 unless otherwise stated, group sizes were determined by power analyses and guided by results of previous studies. Regression analysis was performed by Pearson’s correlation. Data were considered significant if the *P* values were <0.05.

## Results

### Culture of OEhMSCs and MOSJ OS cells in the RWV on collagen I-coated beads

**Fig. 1:**
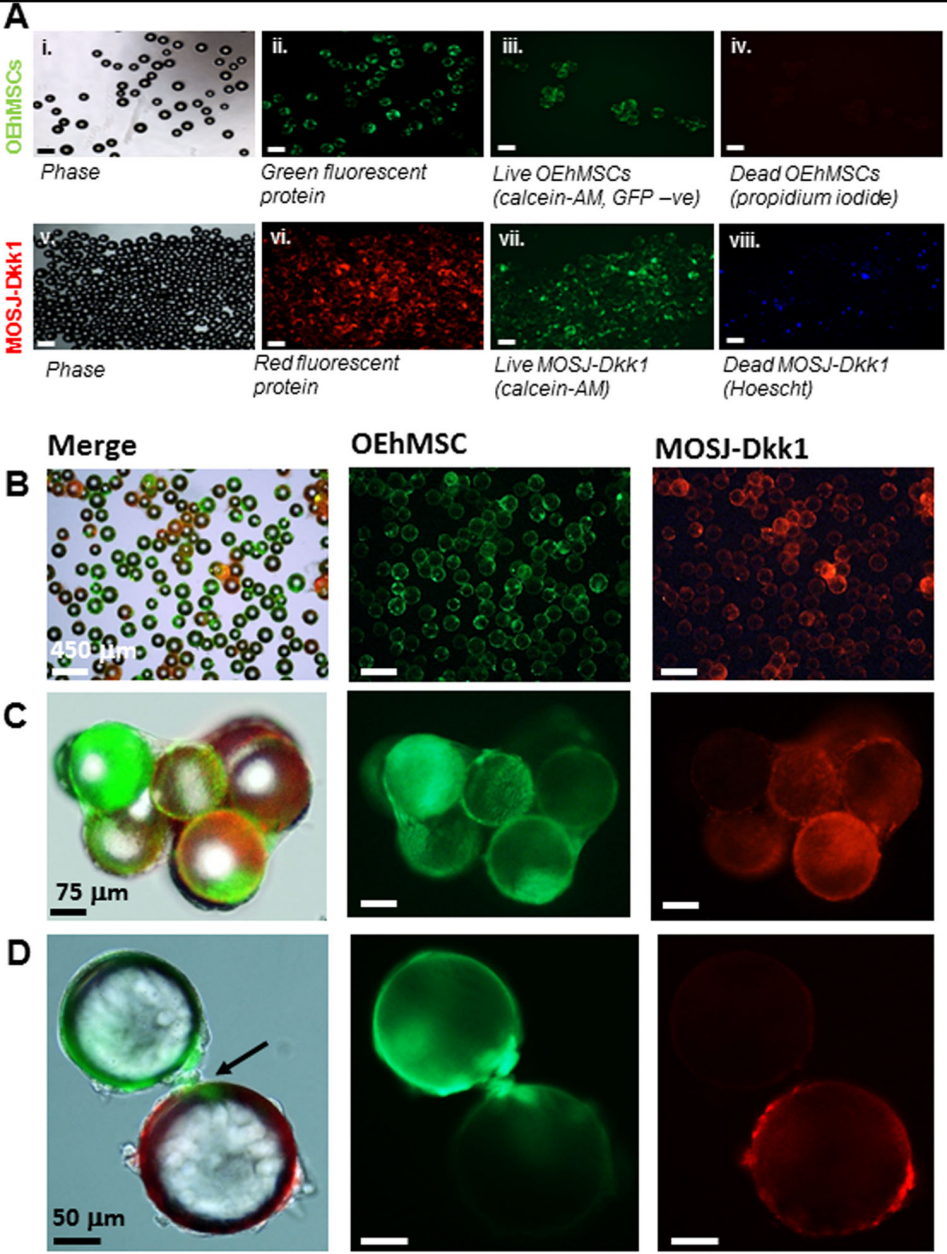
Microcarrier bead -culture of OEhMSCs and MOSJ cells in a RWV: **a** Cells attached to collagen I-coated beads before co-culture in the RWV. Micrographs i–iv illustrate beads loaded with OEhMSCs, micrographs v–viii illustrate beads loaded with MOSJ-Dkk1 cells. Phase (i, v), GFP (ii), calcein AM staining for non-GFP-labeled OEhMSC live cells (iii), and staining of dead cell nuclei (iv). Phase (v), RFP (vi), calcein AM staining for live MOSJ cells (vii) and Hoescht staining of dead MOSJ nuclei (iv). **b–d** Co-cultures of OEhMSCs and MOSJ-Dkk1 cells on collagen I-coated beads. Low- (**b**), mid- (**c**), and high-power (**d**) images indicating presence of RFP-labeled MOSJ-Dkk1 (right) and GFP-labeled OEhMSCs (center), with both merged with phase images (left). High-power micrograph in **d**, indicates (arrowed) OEhMSC-laden bead with an OEhMSC forming a bridge with a MOSJ-Dkk1 laden bead

OEhMSCs from human bone marrow were selected to mimic the osteogenic niche because the cells undergo osteogenesis in vivo^28,30,36,37^. Green fluorescent protein (GFP)-labeled bone marrow-derived hMSCs were characterized according to standard methods (Fig. S1) ^38^. Red fluorescent protein (RFP)-labeled OS cells expressing Dkk-1 (MOSJ-Dkk1) or control OS cells (MOSJ-pLenti) were employed to mimic osteolytic and osteogenic MBD respectively^26^. OEhMSCs and MOSJ cells were first attached to collagen I coated polystyrene beads by incubation with orbital shaking (Fig. 1a). Calcein-AM and propidium iodide or Hoescht staining (live/dead staining) confirmed that the majority of the cells attached to the beads were alive (Fig. 1a). Data for MOSJ-pLenti, cells were the same as those presented for MOSJ-Dkk1.

The experimental rationale is provided in Fig. S2. The hMSCs and MOSJ cells were added separately over a 48 h period to facilitate acclimatization and osteogenic enhancement media (OEM) was then added to the cultures to differentiate hMSCs to OEhMSCs. During the first 1–2 days of culture, the beads homogeneously distributed themselves in the media. After day 3 of co-culture, OEhMSCs and MOSJ cell-laden beads began to aggregate, resulting in clusters of beads up to 4 mm in diameter (Fig. S2D). During early stages of co-culture, OEhMSCs and MOSJ cells remained on their respective beads (Fig. 1b), but as aggregation began to occur, we observed transfer of cells between the beads (Fig. 1c). While the directionality of the transfer was unclear, we observed several instances where OEhMSCs appeared to migrate by establishing attachment points bridging beads (Fig. 3d). While bridging by MOSJ cells was not observed, MOSJ cells were present on the majority of beads with OEhMSCs, indicating that they had transferred by some means.

**Fig. 2:**
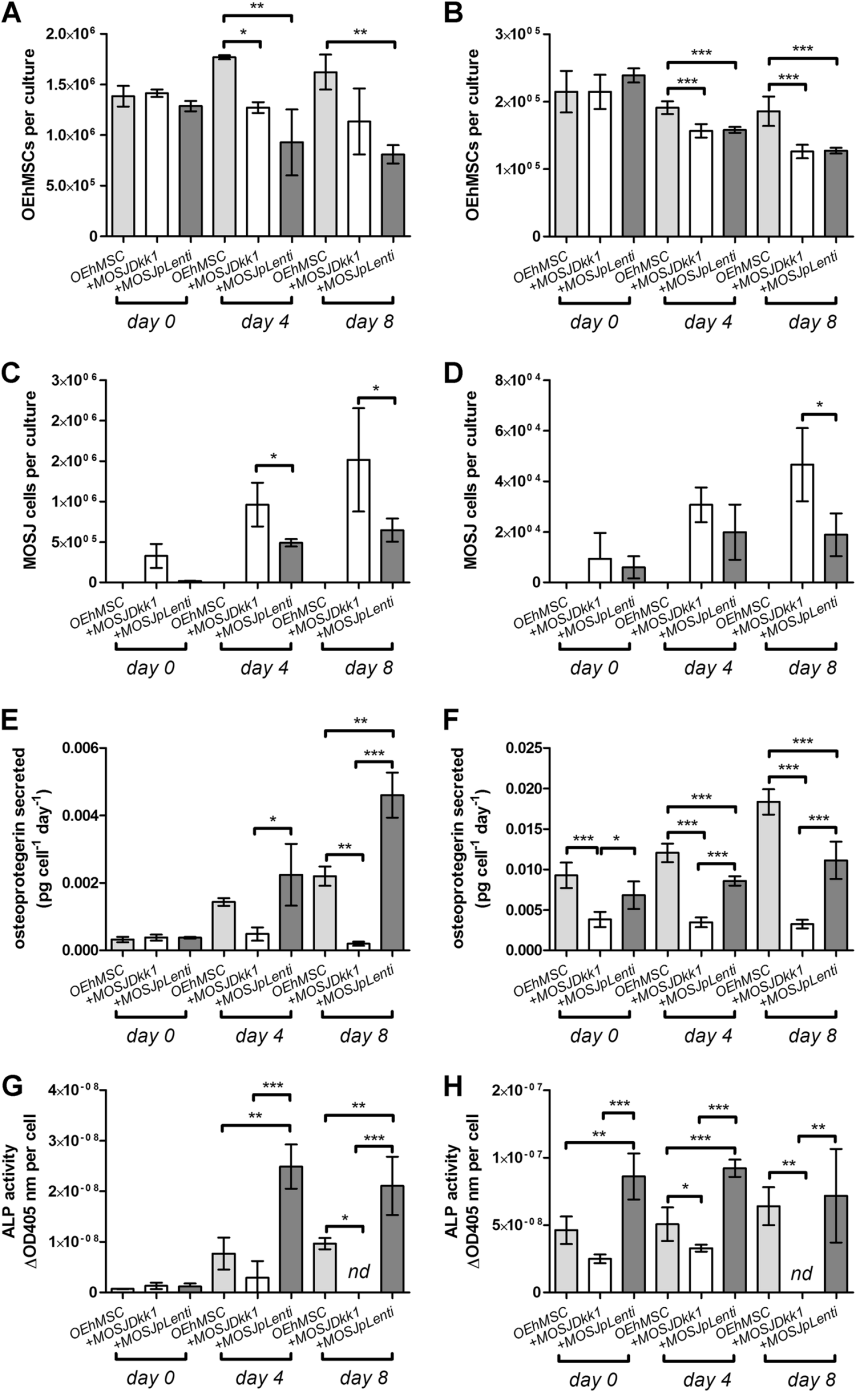
Osteogenic activity of OEhMSCs cultured in the presence and absence of MOSJ-Dkk1 or MOSJ-pLenti cells on collagen I. **a**, **c**, **e**, **g** RWV co-cultures. **b**, **d**, **f**, **h** Monolayer co-cultures. **a**, **b** Enumeration of OEhMSCs (refers to 50 cm^2^ growth area). **c**, **d** Enumeration of MOSJ cells (refers to 50 cm^2^ growth area). **e**, **f** Secretion of OPG as measured by ELISA. **g**, **h** ALP activity by OEhMSCs cultured for up to 8 days in the presence or absence of MOSJ cells. The label “*OEhMSC*” refers to OEhMSC monoculture and “*+* *MOSJ…*” refers to co-culture of MOSJ and OEhMSC cells. Statistics: data presented with means and standard deviations. Comparisons were ANOVA with Tukey’s post test. *P* < 0.05*, <0.01**, <0.005***

**Fig. 3:**
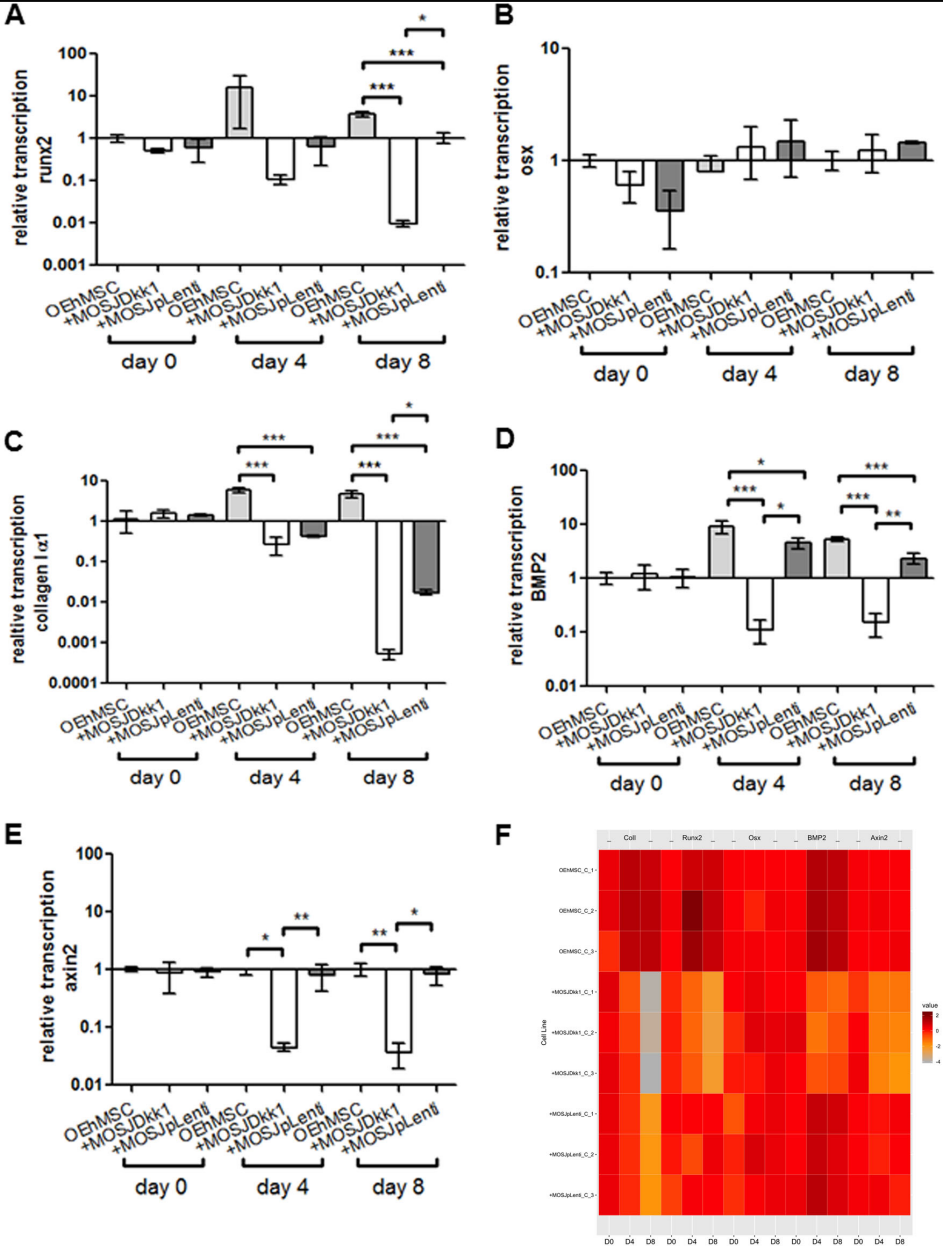
qRT-PCR assays of osteogenic and Wnt-responsive transcripts in RWV co-cultures with collagen I-coated beads. Human-specific qRT-PCR was performed for osteogenic transcripts runx2 (**a**), osterix (**b**), collagen Iα1 (**c**) BMP2 (**d**) and Wnt-responsive transcript axin2 (**e**). Values are presented as transcription relative to OEhMSC-only control cultures where the expression level is set to 1. **f** Heat map comparison of data in **a**–**e** with individual measurements indicated. Statistics: Statistical treatment as in Fig. 2

### Osteogenic activity of OEhMSCs in co-cultures with MOSJ cells on collagen I coatings

In contrast with the parental line, MOSJ-Dkk1 cells exhibit potent osteoinhibitory and osteolytic properties^26^. We expected that the osteoinhibitory mechanisms of Dkk-1 could be recapitulated through co-culture with OEhMSCs, but initial experiments were performed to confirm that OEhMSCs underwent osteogenesis in the RWV. Over an 8-day culture period, OEhMSC cell numbers remained stable, but cell recoveries were significantly higher from RWVs than from monolayers (Fig. 2a, b, Fig. S4A&B). Secretion of the osteogenic ligand osteoprotegerin (OPG) rose in each culture system, but final levels were lower in RWVs than in monolayers (Fig. 2e, f). Likewise, when the osteogenic marker alkaline phosphatase (ALP) was assayed, activity was higher in monolayer cultures than RWV cultures at day 8 (Fig. 2g, h). While RWV cultures resulted in lower overall readouts in the osteogenic assays, the signals in the RWV were robust and the transition from hMSC to OEhMSC was more apparent in RWVs as compared to monolayers. This was due to significantly lower baseline OPG and ALP levels, suggesting that RWV culture does not permit premature osteogenic differentiation of hMSCs, a phenomenon often observed in monolayers. Osteogenic differentiation by OEhMSCs in RWVs was further supported by increased transcription of the master regulator of osteogenesis, runt-related transcription factor 2 (Runx2) (Fig. 3a), type I collagen (Fig. 3c) and bone morphogenic protein 2 (BMP2) (Fig. 3d). Transcription of the osteogenic transcription factor osterix (OSX) was unaffected (Fig. 3b).

Upon co-culture with OEhMSCs, MOSJ-Dkk1 cells proliferated strongly in both systems, with a reduction in the number OEhMSCs, presumably due to competition for nutrients and attachment (Fig. 2a, b, Fig. S5C&D, Fig. S6A&B). As predicted, MOSJ-Dkk1 cells had a profound effect on osteogenesis by OEhMSCs, reducing OPG and ALP in both culture systems, but OEhMSCs in monolayer cultures exhibited a greater degree of resistance to the osteoinhibitory stimuli (Fig. 2e–h). Transcription of Runx2, collagen I, and BMP2 were reduced as compared to OEhMSCs cultured alone (Fig. 3a, c, d). In agreement with the hypothesis that Dkk-1 inhibits osteogenesis by targeting cWnt signaling, Axin2, a reporter for the pathway^39^, was significantly downregulated as compared to OEhMSCs cultured alone (Fig. 3e).

The control cells for MOSJ-Dkk1 (MOSJ-pLenti) are comparatively osteogenic^26^. Upon co-culture with OEhMSCs, MOSJ-pLenti cells proliferated, but generally to a lesser degree than MOSJ-Dkk1 cells, also resulting in loss of OEhMSCs (Fig. 2a–d, Fig. S5E&F, Fig. S6C&D). In both culture systems, there was no robust sign of osteogenic inhibition with MOSJ-pLenti cells (Fig. 2e–h, Fig. 3). Axin2 levels were also unchanged (Fig. 3e), suggesting that cWnt had not been affected by co-culture with MOSJ-pLenti cells.

These data demonstrate that hMSCs have the capacity to differentiate into OEhMSCs in the RWV bioreactor and that this process is inhibited by MOSJ-Dkk1 cells, but not control MOSJ-pLenti cells.

### Processing of ECM and generation of ECM-coated beads

OEhMSCs generate ECM that contains factors present in anabolic bone tissue^28,30^ that support the growth of bone^30,37^. A superior recapitulation of the osteogenic niche may therefore be mimicked by coating the beads with OEhMSC-derived ECM. OEhMSC-derived ECM (hereafter ECM) was generated from OEhMSCs^28,30^. After processing, the ECM was acellular and fibrous (Fig. S4A&B) and SEM with hMSCs confirmed that the ECM supported cell attachment (Fig. S4B). OEhMSCs, but not undifferentiated hMSCs, secreted greater amounts of OPG when cultured on ECM-coated monolayers, as compared to a mixture of ECM:collagen I and collagen I alone (Fig. S4C). ECM was covalently attached to beads using EDAC-mediated coupling (Fig. S4D). SEM demonstrated that aggregates of ECM attached to the beads (Fig. S4E) and immunostaining for collagen I also confirmed that attachment had taken place (Fig. S4F).

### Osteogenic enhancement of OEhMSCs when attached to ECM-coated beads

To investigate whether the ECM-coated beads could enhance osteogenesis, we cultured hMSCs on ECM-coated beads in OEM. Similar experiments were performed with collagen I-coated beads for comparison. Transcriptome profiling was performed after 8 days of culture yielding 631 transcripts that were upregulated on ECM as compared to collagen I by more than two-fold. Gene ontology clustering highlighted genes responsible for cell attachment and adhesion signaling, suggestive of signal transduction through ECM (Table 1A). When the list was compared with known tissue expression signatures, bone and bone marrow exhibited the highest enrichment scores (Table 1B), indicating that the genes upregulated on ECM (as compared to collagen I) were more closely associated with bone and bone marrow rather than other tissue types in the database.

**Table 1:** Analyses of genome profiles generated by OEhMSCs cultured in the RWV on beads coated with OEhMSC-derived ECM as compared to collagen I

A		
Gene ontology		
Term	*P*-value	Enrichment
Response to mechanical stimulus	1.90E−06	7.4
Integrin binding	2.90E−04	4.2
Cellular response to hypoxia	7.00E−04	4.1
Positive regulation of cell migration	2.40E−04	3.2
Positive regulation of gene expression	1.10E−05	3.2
ECM organization	4.60E−04	3
Proteinaceous ECM	3.80E−05	3
Aging	3.10E−03	2.9
Cell–cell adhesion	1.90E−04	2.8
Cadherin binding involved in cell–cell adhesion	1.30E−04	2.8
Heparin binding	6.90E−03	2.8
ECM	1.40E−04	2.7
Mitochondrial outer membrane	1.10E−02	2.7
Angiogenesis	1.60E−03	2.7
Focal adhesion	4.10E−05	2.6
B		
Tissue expression		
**Term**	***P*** **-value**	**Enrichment**
Bone^a^	1.40E−08	4
Bone marrow^b^	8.40E−11	3.9
Ovary	3.30E−10	3.7
Eye	2.10E−08	3.7
Brain	1.80E−09	3.7
Lung	9.40E−08	3.4
Eye	4.70E−10	3.3
Vascular	1.90E−05	2.5
Thyroid	2.10E−04	2.3

^a,b^ refer to Table S2 and 3, respectively

A list of those transcripts with 2-fold greater transcription when hMSCs differentiated to OEhMSCs on ECM as compared to on collagen I was subjected to analysis using the DAVID database. A: Gene ontology clustering. B: Association with known tissue expression signatures. The definition of *P*-values and Enrichment Scores are provided in Huang da et al.^31,32^

### Osteogenic activity of OEhMSCs in co-cultures with MOSJ cells on ECM

**Fig. 4:**
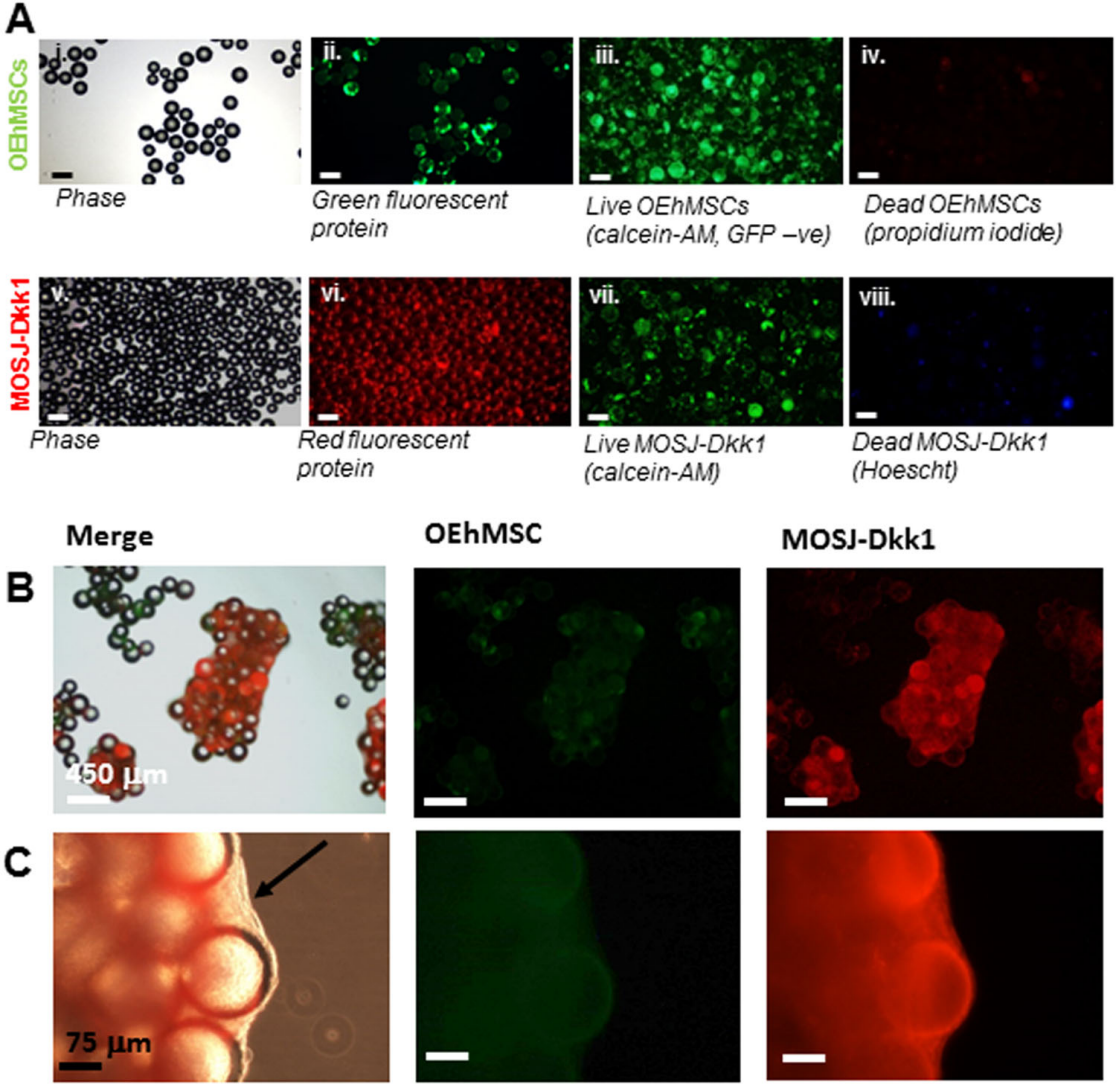
Attachment and co-culture of OEhMSCs and MOSJ cells on OEhMSC-derived ECM-coated beads. **a** Cells attached to ECM-coated beads before co-culture in the RWV. Micrographs i–iv illustrate beads loaded with OEhMSCs, micrographs v–viii illustrate beads loaded with MOSJ-Dkk1 cells. Phase (i), GFP (ii), calcein AM imaging of non-GFP-labeled live OEhMSCs (iii) and propidium iodide staining of dead cell nuclei (iv). Phase (v), RFP (vi), calcein AM staining for live cells (vii) and Hoescht staining of dead cell nuclei (viii). **b,** **c** Co-cultures of OEhMSCs and MOSJ-Dkk1 cells on ECM-coated beads. Low- (**b**), and high-power (**c**) images indicating abundance of RFP-labeled MOSJ-Dkk1 (right) and sparse levels of GFP-labeled OEhMSCs (center) with both merged with phase image (left). Panel **c** illustrates presence of densely packed clusters of MOSJ-Dkk1 cells held together by ECM (arrowed)

**Fig. 5:**
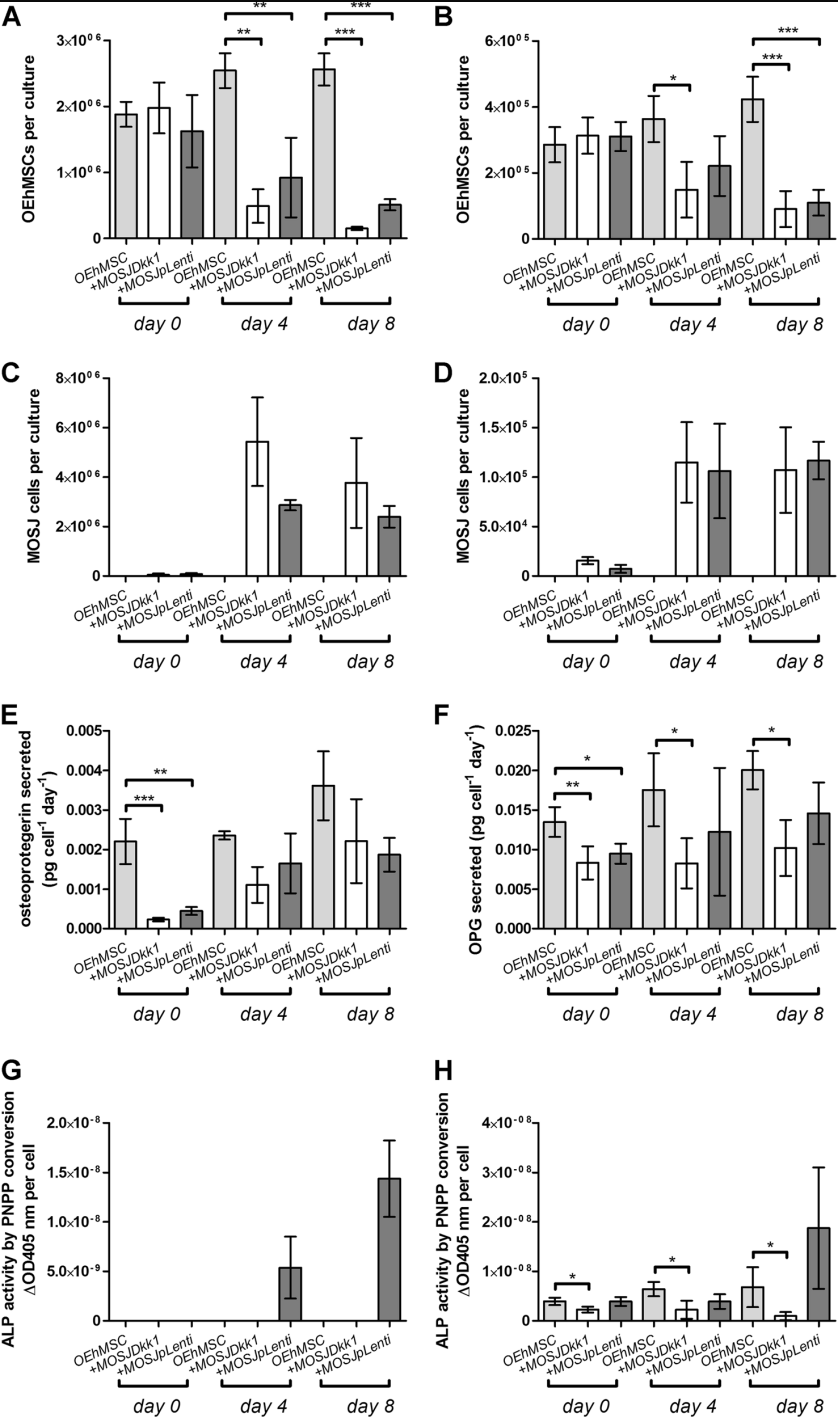
Osteogenic activity of OEhMSCs cultured in the presence and absence of MOSJ-Dkk1 or MOSJ-pLenti cells on ECM: **a**, **c**, **e**, **g** RWV co-cultures. **b**, **d**, **f**, **h** Monolayer co-cultures. **a**, **b** Enumeration of OEhMSCs (refers to 50 cm^2^ growth area). **c**, **d** Enumeration of MOSJ cells (refers to 50 cm^2^ growth area). **e**, **f** Secretion of OPG as measured by ELISA. **g**, **h** ALP activity by OEhMSCs cultured for up to 8 days in the presence or absence of MOSJ cells. The label “*OEhMSC*” refers to OEhMSC monoculture and “*+MOSJ…*” refers to co-culture with MOSJ and OEhMSC cells. Statistical treatment as in Fig. 2

OEhMSCs were co-cultured with MOSJ cells on ECM-coated beads. When visualized by microscopy, OEhMSCs, MOSJ-Dkk1 cells, and MOSJ-pLenti cells attached to the beads and established viable cultures (Fig. 4a). As observed with collagen I coating, the cultures began to generate aggregates but resulted in increased OEhMSC yields as compared to collagen I coated beads in both culture systems (Fig. 5a, b, Fig. S5A&B).

When OEhMSCs were cultured with both MOSJ subtypes, MOSJ cells rapidly proliferated, generating aggregates that were held together by a dense matrix (Fig. 4b, c). At days 4 and 8, OEhMSCs numbers were reduced by co-culture with either MOSJ subtype on ECM in both culture systems (Fig. 5a, b, Fig. S5C-F). In RWV cultures, the negative effect of MOSJ cells on OEhMSC recoveries was ECM-dependent since it was not observed with collagen I (Fig. S5C). Together, these data demonstrate that ECM coatings result in a pronounced increase in the rate of MOSJ proliferation, resulting in rapid displacement of OEhMSCs.

A reduction of OPG was evident in co-cultures containing both MOSJ subtypes, especially early in culture. However, at days 4 and 8, MOSJ-Dkk1 co-cultures generated a surprisingly comparable amount of OPG as compared to control and MOSJ-pLenti and control conditions (Fig. 5e, f), suggesting that attachment to ECM could be rescuing the OPG secretion by OEhMSCs to some degree. With respect to ALP activity, monolayers with ECM coatings were similar to collagen I coatings (Fig. 5h). Surprisingly, ALP could only be detected in RWV co-cultures containing MOSJ-pLenti cells suggesting that ECM contributed to inhibition of late stage osteogenic markers such as ALP (Fig. 5g).

**Fig. 6:**
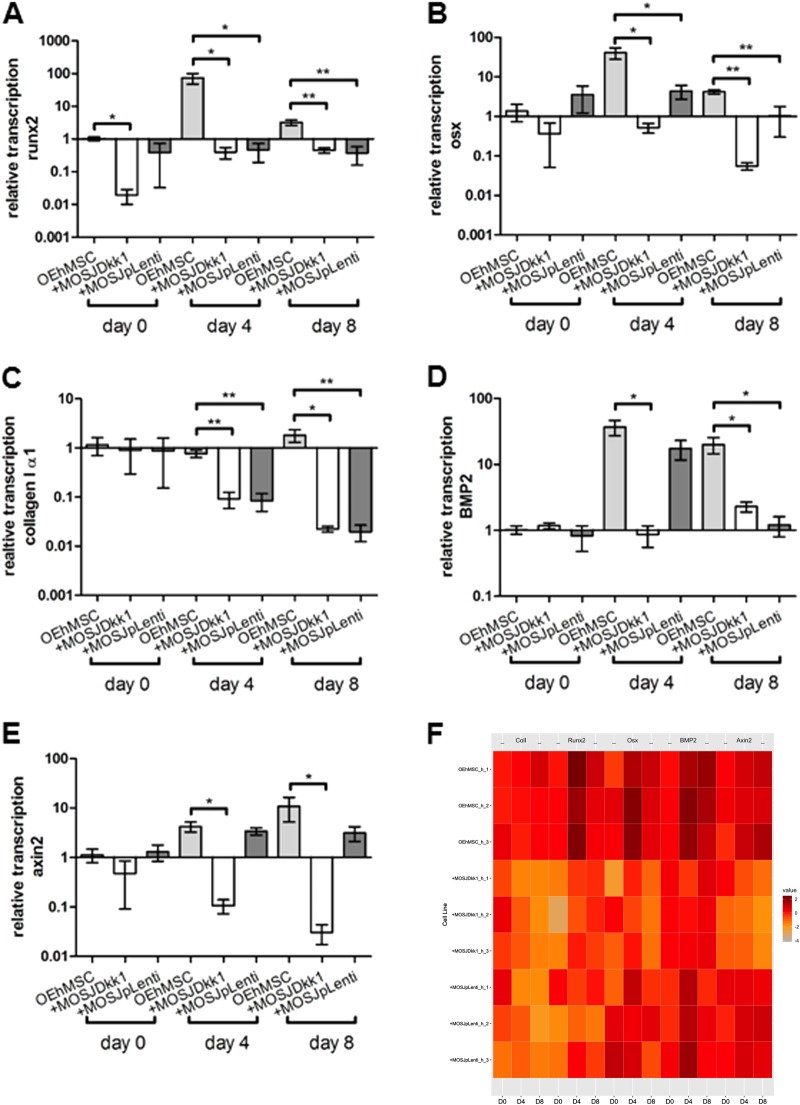
qRT-PCR assays of osteogenic and Wnt-responsive transcripts in RWV co-cultures with ECM-coated beads. Human-specific qRT-PCR was performed for osteogenic transcripts runx2 (**a**), osterix (**b**), collagen Iα1 (**c**) BMP2 (**d**) and Wnt-responsive transcript axin2 (**e**). Values are presented as transcription relative to OEhMSC-only control cultures where the expression level is set to 1. **f** Heat map comparison of data in **a**–**e** with individual measurements indicated. Statistics: Statistical treatment as in Fig. 2

The transcription of early osteogenic transcription factors, Runx2 and OSX by OEhMSCs was measured by qRT-PCR (Fig. 6a, b). In the absence of MOSJ-cells, both transcription factors were upregulated with growth on ECM by about 100-fold and 50-fold, respectively, as compared to approximately 10-fold and 1-fold respectively on collagen I during osteogenic stimulus. The osteogenic ligand, BMP2 was also upregulated on ECM by approximately 50-fold at day 4 and day 8 as compared to 8–10 fold on collagen I (Fig. 6d). Together, these data support the hypothesis that ECM upregulates early markers of osteogenic differentiation. Co-culture with either MOSJ cell type reduced transcription of Runx2, OSX, collagen I and BMP2 (Fig. 6a–d) as compared to controls. Axin2 expression was downregulated in OEhMSCs cultured in the presence of MOSJ-Dkk1 cells, confirming that MOSJ-Dkk1 cells were inhibiting cWnt signaling (Fig. 6e).

### Growth of PDX fragments in the RWV

**Fig. 7:**
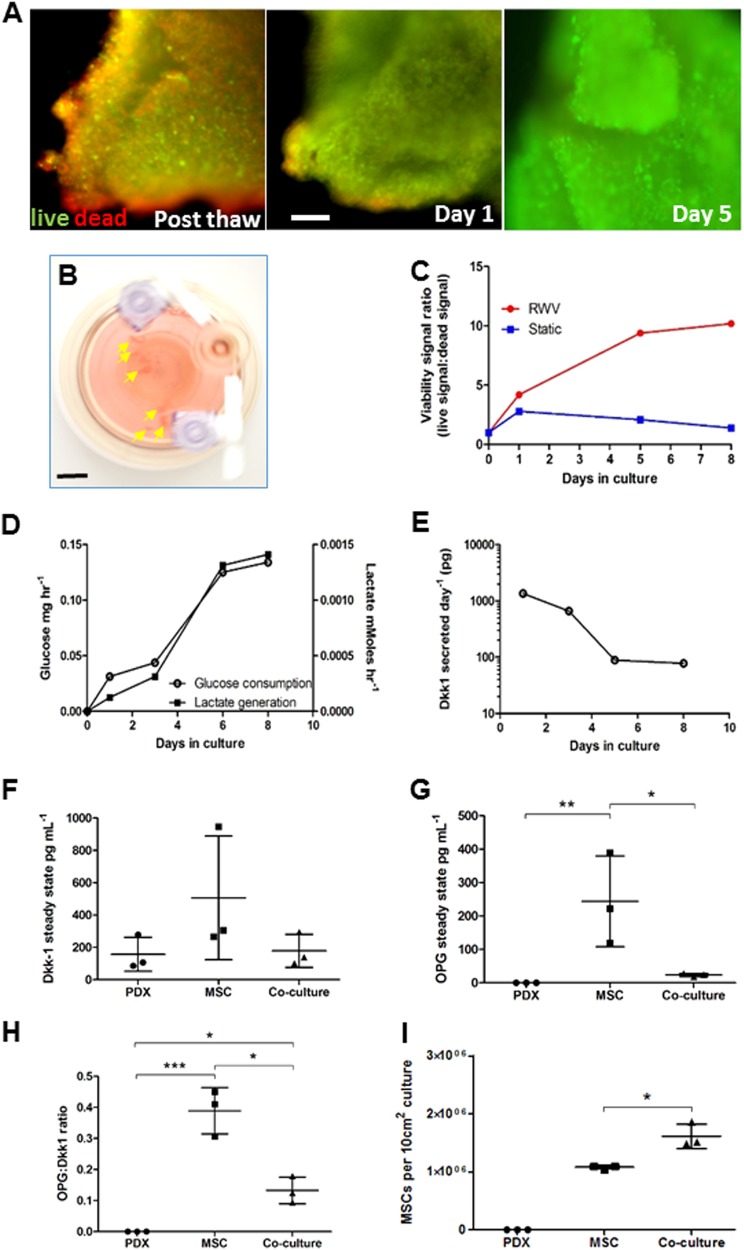
Growth of PDX specimens in the RWV. **a** Fluorescence micrographs of live/dead-stained PDX fragments immediately after recovery from cryopreservation (Post thaw) and after RWV culture. The green channel indicates live CalceinAM-positive cells and the red channel indicates dead propidium iodide-positive dead cells (bar = 100 μm). **b** RWV cultures containing OS PDX fragments (arrowed) (bar = 10 mm). **c** Viability status of PDX specimens as a ratio of green fluorescence intensity (live) to red fluorescence intensity (dead) over time in RWV culture or static culture. Typical result from 3 × 4 mm^3^ fragments. Experiment was performed on two individual tumor specimens. **d** Glucose consumption and lactate generation by OS PDX cultures over time. Experiment was performed on two individual tumor specimens. **e** Dkk-1 output over time in RWV culture as measured by ELISA on media samples. **f** Dkk-1 output by 5 × 1 mm^3^ PDX fragments, OEhMSC-laden collagen-coated beads or co-cultures as measured by ELISA on 2-day conditioned media. **g** As in **f**, but measurements of OPG. **h** As in **f**, but ratios of OPG:Dkk-1 measurements. **i** Yields of OEhMSCs recovered from RWV cultures. Statistics: for **c**–**e**, data are means of six technical replicates from a representative of four cultures. For **f**–**i**, *n* = 3 and values are expressed as means and standard deviations, points represent individual measurements. Statistical treatment as in Fig. 2

PDX specimens previously shown to express Dkk-1 in vivo^35^ were morselized and cultured in the RWV. After recovery from cryopreservation, PDX fragments harbored a significant number of dead cells, but after 5 days in the RWV, virtually no dead cells could be observed (Fig. 7a). The PDX fragments increased in volume (Fig. 7b) while increasing glucose consumption and lactate output (Fig. 7d). Dkk-1 secretion was highest between days 1 and 5 of culture, dropping to approximately 1 tenth of maximum by day 8 (Fig. 7e). In contrast with RVW culture, viability of PDX fragments in static culture deteriorated (Fig. 7c).

To investigate whether PDX fragments could affect osteogenic differentiation, they were co-cultured with hMSCs attached to collagen I coated beads under osteogenic conditions. Attempts to separate the OEhMSCs from PDX tissue failed, preventing the assay of OEhMSC differentiation without interference from PDX cells. Nevertheless, ELISA assays of Dkk-1 and OPG in the media were possible. Surprisingly, Dkk-1 levels were lower in PDX cultures as compared to OEhMSCs cultured alone, but this apparent difference was not significant due to variability in Dkk-1 output by replicates (Fig. 7f). OPG was absent in PDX cultures and high in cultures of OEhMSC alone. When PDXs were co-cultured with OEhMSCs, OPG output dropped, indicating that the PDX samples had osteoinhibitory potential (Fig. 7g). When ratios of secreted OPG to Dkk-1 levels were calculated and plotted, the effects of variability were substantially reduced and the trend was robustly maintained (Fig. 7h). To exclude the possibility that OPG:Dkk-1 ratios were attributable to variable OEhMSC levels, the cells were enumerated. OEhMSCs in both OEhMSC-only and co-cultures were comparable with a statistically significant increase in co-cultures (Fig. 7i). Collectively, these results confirmed that Dkk-1 positive PDX tumors exhibited osteoinhibitory effects in the co-culture system.

## Discussion

A minimal recapitulation of the osteogenic niche requires osteoprogenitors, the correct attachment substrate, and a 3D environment. Growth of OEhMCSs in the RWV represented an effective solution because OEhMSCs have been shown to regenerate bone in vivo^30,36^ and RWVs have the potential to support large 3D structures^18,19^. Beads are readily available, have predictable characteristics in the RWV, and when aggregated, provide a mimic of the trabecular architecture of bone tissue^40–42^. An OS cell line engineered to express Dkk-1 (MOSJ-Dkk1) was chosen to test the system because Dkk-1 has been implicated as a potent contributing factor in several forms of lytic MBD^9^. As compared to the control MOSJ line that does not express high levels of Dkk-1, MOSJ-Dkk1 cells generate larger tumors with strong lytic activity in vivo^26^.

Initially, co-cultures were performed on a commercial collagen I coated bead and we found that over 8 days, the yield of OEhMSCs from RWVs was significantly greater than from monolayers. RWV culture also facilitated the formation of 3D aggregates, a characteristic not attainable by monolayer culture^43^. Reduced osteogenic differentiation in the simulated microgravity of RWV bioreactors^44–46^ and microgravity itself^47–49^ has been reported in the literature. We also observed that osteogenic differentiation in the RWV was reduced as compared to monolayer culture. However, while the magnitude of measurements for the osteogenic assays were lower for RWVs as compared to monolayers, the progression of differentiation was more apparent in the RWVs due to the lower level of basal activity. In short, we measured an incremental increase in osteogenic capacity from a high baseline with monolayers, whereas a robust osteogenic signal from a near-zero baseline was observed in RWVs.

The use of the RWV provides the opportunity to study transfer of cells from one attachment site to another. Upon initiation of co-cultures, the OEhMSCs and MOSJ cells were seeded on two separate populations of beads, but the distribution of cells rapidly became homogeneous, suggesting bidirectional transfer. The kinetics and mechanism of this phenomenon is unclear, but in some cases, hMSCs appeared to be attached to two beads simultaneously, suggesting migration through cell motility. While not directly observed in the experiments, transfer between beads through detachment and reattachment is also possible^50^, and more likely for tumor cells given their aptitude for extended survival without attachment. The RWV co-culture system therefore shows promise for the study of tumor cell engraftment.

We have reported that OEhMSC-derived ECM enhances osteogenesis^28,30,36,37^. When OEhMSCs were grown on the ECM under osteostimulatory conditions in the RWV, the ECM accelerated osteogenesis by hMSCs. The results also suggested that ECM appeared to preferentially upregulate the expression of early markers of osteogenesis such as OPG, Runx2 and BMP2 when compared to OEhMSCs cultured on collagen I. OSX expression increased on the ECM-coated beads but not the type I collagen-coated beads. The reasons for these observations are unclear, but OSX functions downstream of Runx2 in the osteoblast differentiation pathway^51^ and these data may be the result of a more comprehensive osteogenic stimulus provided by the ECM, especially when one considers the relatively limited signalling capacity of purified collagen I. Similarly, the reduced rate of collagen I expression by OEhMSCS on ECM-coated beads is probably a feedback effect in response to the presence of a more comprehensive repertoire of ECM factors. On pure collagen I, OEhMSCs will detect a deficit of ancillary collagens that are present in heterotypic fibrils and collagen I will be co-expressed with these collagens so as to assemble the fibrils correctly. On a complex ECM, many of these collagens are present, and this process is expected to be downregulated.

Surprisingly, ECM inhibited ALP activity as compared to collagen I. One explanation for this observation is that that ALP activity plays a key role in mineralization^52^ and presence of trace calcium phosphate in ECM^28^ may feedback to inhibit further mineralization and associated ALP activity by the OEhMSCs. Furthermore, the data presented herein demonstrate that the ECM maintains an early osteogenic phenotype, as evidenced by upregulated Runx2, OSX, BMP2 and the upregulation of canonical Wnt signaling which has been shown to drive early commitment to osteoblasts^53^. Previously we have shown that OEhMSCs promote bone healing in vivo and that prolonging the early osteogenic phase by attachment to ECM can prolong the activity of the OEhMSCs and enhance bone formation^28,30,37^. Collectively, these data suggest that early markers of bone formation may be more accurate predictors of osteogenic capacity.

Upon co-culture of MOSJ cells with OEhMSCs on ECM, we measured a surprisingly rapid accumulation of MOSJ cells that displaced OEhMSCs. Under conditions of RWV culture and ECM coating, proliferation of overwhelming numbers of MOSJ cells coupled with the formation of a dense matrix, could serve as the major osteoinhibitory mechanism, rather than the more specific action of Dkk-1 as is seen on collagen I coatings. The measurable inhibition of osteogenesis by Dkk-1 on collagen I suggest that this approach is useful for studying subtle mechanisms that could be complicated by excessive tumor cell overgrowth, whereas utilization of ECM is more suited to studies that require aggressive formation of 3D tumor-like structures.

The role of Dkk-1 and cWnt on osteogenesis has been demonstrated by in vitro studies,^3,9,10,54^ and these data have provided a strong rationale for the targeting of Dkk-1 for treatment of OLs. Surprisingly few co-culture experiments have been performed focusing on the interaction of Dkk-1 expressing tumors and osteoprogenitors even though the presence of stroma can profoundly affect proliferation and viability of tumor cells^3,55^. Furthermore, tumors can modulate the behaviour of osteoprogenitors through secretion of bioactive factors and competition for attachment sites and nutrients. In this study, co-cultures of OEhMSCs with OS cells clearly demonstrated that Dkk-1 expression has an inhibitory effect on osteogenesis, but we also demonstrate that competition for attachment sites their composition likely play additional roles in the pathology of MBD.

In the RWV, PDX fragments were metabolically active, increased in volume, and recovered viability after thawing. This approach is likely to be valuable for the generation of research material, and provides an animal-free and simplified platform. Moreover, it raises the attractive possibility of rapidly performing multiple drug sensitivity trials and molecular analyses on low-volume tumor biopsies.

When human PDX specimens were co-cultured with OEhMSCs, the osteogenic status of the co-culture could be determined through measurements of Dkk-1 and OPG. In these experiments, Dkk-1 levels were found to be relatively high and variable when OEhMSCs are cultured alone. This observation is likely to be due to the sensitivity of Dkk-1 expression to local variations in MSC density^56^. In contrast, OPG was high in cultures of OEhMSCs, lower in co-cultures, and not detectable in cultures of PDX cultures alone. To improve the robustness of the system, we postulated that ratios of OPG to Dkk-1 output could represent a biologically meaningful measure of the osteogenic status of the cultures. The premise for this approach is supported by the observation that bone is homeostatically regulated by cWnt signalling with Dkk-1 and OPG representing opposing osteoinhibitory and osteoinductive stimuli respectively^57^. As predicted, OPG:Dkk-1 ratios were high in OEhMSCs cultured alone, zero for PDX cultures, and significantly lower than OEhMSC controls for co-cultures. We also observed a slight increase in OEhMSC yields when co-cultured with PDX cells suggesting that in the absence of competition for growth area, PDX co-cultures can exert trophic effects on the surrounding stroma. Given that tumor-associated stroma has, in turn, the capacity to provide tumor survival factors, recapitulation of this phenomenon in vitro could facilitate the study of tumor-stroma interactions and the development of novel therapies that may disrupt this process. In these experiments, collagen-coated beads rather than ECM-coated beads were used in co-cultures of OEhMSCS and PDX fragments so as to minimize the possibility of PDX cells or cell clusters engrafting and displacing the OEhMSCs, a phenomenon observed with MOSJ co-cultures. In subsequent studies, the effect of ECM versus collagen I on engraftment of PDX-derived cells could uncover mechanisms of tumor cell engraftment and metastasis.

Herein, we describe the utilization of the RWV bioreactor for the modelling of bone-tumor interactions. The use of the RWV to model bone–tumor interactions provided several advantages over standard monolayer culture, such as substantially increased cell yields, a closer mimic of the topology of bone tissue, and lower baseline osteogenic signals, but it also provided the opportunity to study transfer of cells from one attachment site to another and introduced a feasible approach for the comparison of different attachment substrates. Culture of intact tumor specimens was also possible, providing the means to expand the specimen or to perform drug susceptibility trials and analyses without the need for implantation in animals. The ability to rapidly culture patient-derived tumor specimens raises the attractive opportunity for the design of patient-specific treatment protocols from small biopsies or surgically recovered material.

## Electronic supplementary material

Supplemental text

Figure S1

Figure S2

Figure S3

Figure S4

Figure S5

Figure S6

## Data Availability

Raw data available on request. Transcriptomic data accepted by Gene Expression Omnibus, awaiting processing (GE118107).
