# Supplementary material for: Three-dimensional in vitro modeling of malignant bone disease recapitulates experimentally accessible mechanisms of osteoinhibition
Source: Cell Death Dis. 2018 Nov 26;9(12):1161. doi: 10.1038/s41419-018-1203-8 (PMC6255770; doi:10.1038/s41419-018-1203-8)
Supplement: Supplementary file 1 — Supplemental text [file 41419_2018_1203_MOESM1_ESM.docx]

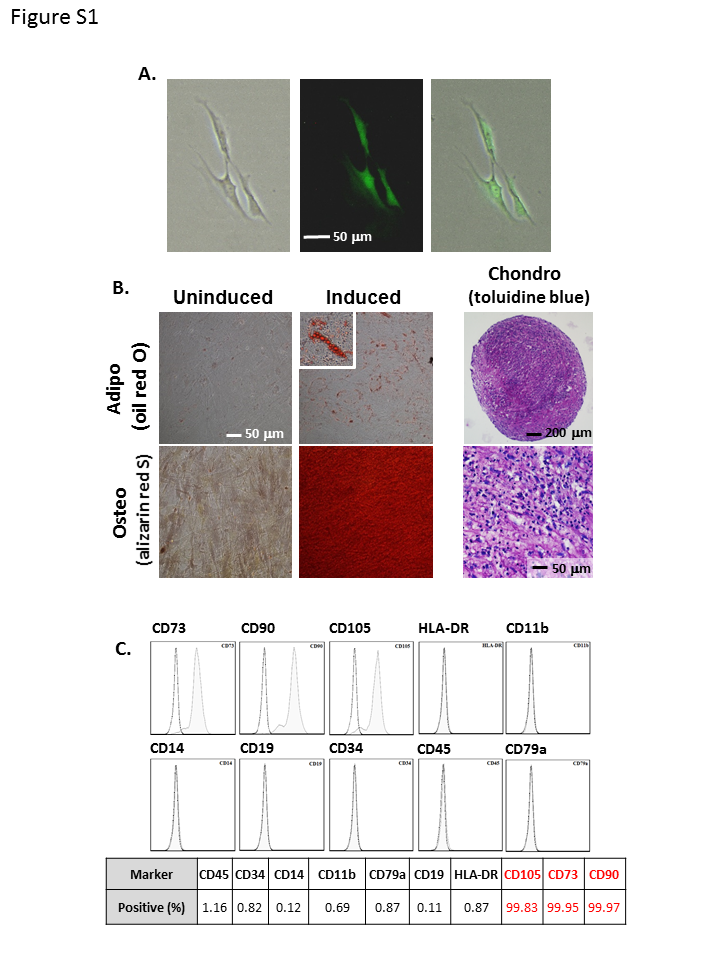


**Figure S1: Characterization of GFP-labeled human mesenchymal stem cells used in the study. Panel A:** Phase, GFP and merged image of GFP-hMSCs used in the study. **Panel B:** Adipogenic monolayer with lipid vacuoles (magnified in *inset*) stained with oil red O (*above center*). Osteogenic monolayer with mineralized matrix stained with alizarin red S (*below center*). Chondrogenic micromass with articular cartilage stained purple using toluidine blue (*far right*). **Panel C:** Flow cytometry confirmed that the hMSCs express the correct immunophenotype as defined by Dominici *et al.* 2006 (1).


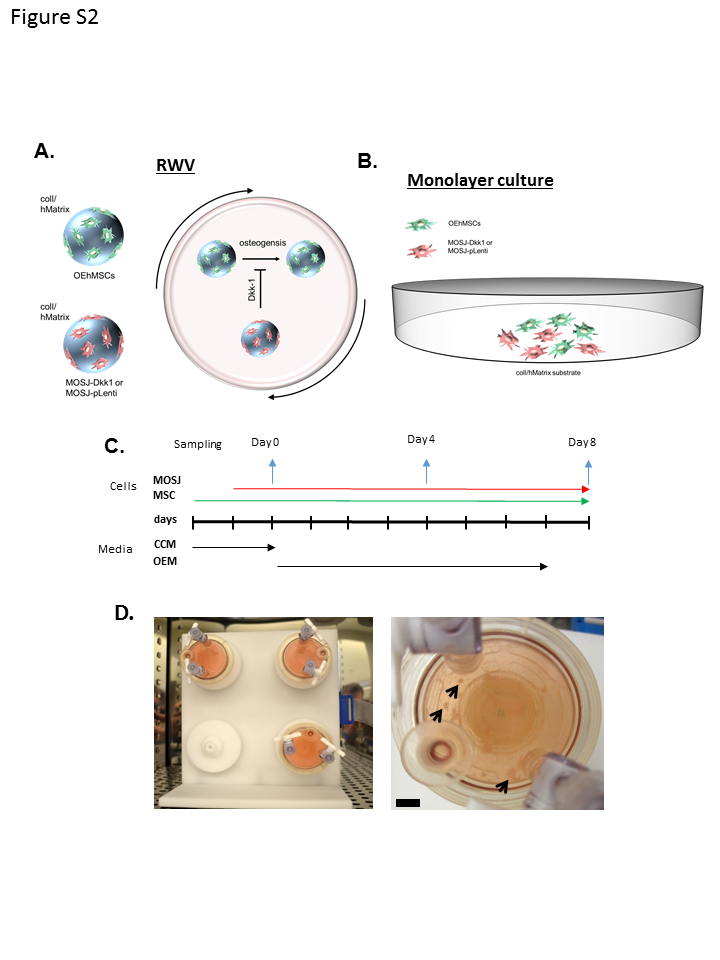


**Figure S2 Experimental setup of RWV and monolayer co-cultures: Panel A-B:** GFP-labeled MSCs and dsRed labeled MOSJ OS cells (MOSJ-Dkk1 or MOSJ-pLenti controls) were loaded onto collagen I or ECM coated polystyrene beads and co-cultured for up to 8 days under osteogenic conditions in a Synthecon RWV system. Equivalent control assays are performed using monolayer cultures. **Panel C:** Time course for media changes and sampling. Cell numbers and osteogenic assays are performed at day 0, day 4 and day 8 post osteogenic induction. CCM: complete culture media. OEM: osteoenhancement media. **Panel D:** RWV set up with 3 10 mL cultures (*left*) and close-up view (*right*) after 8 days of RWV showing loosely aggregated cell-laden beads containing OEhMSCs and MOSJ-Dkk1 cells (*arrowed*) (bar = 10 mm).


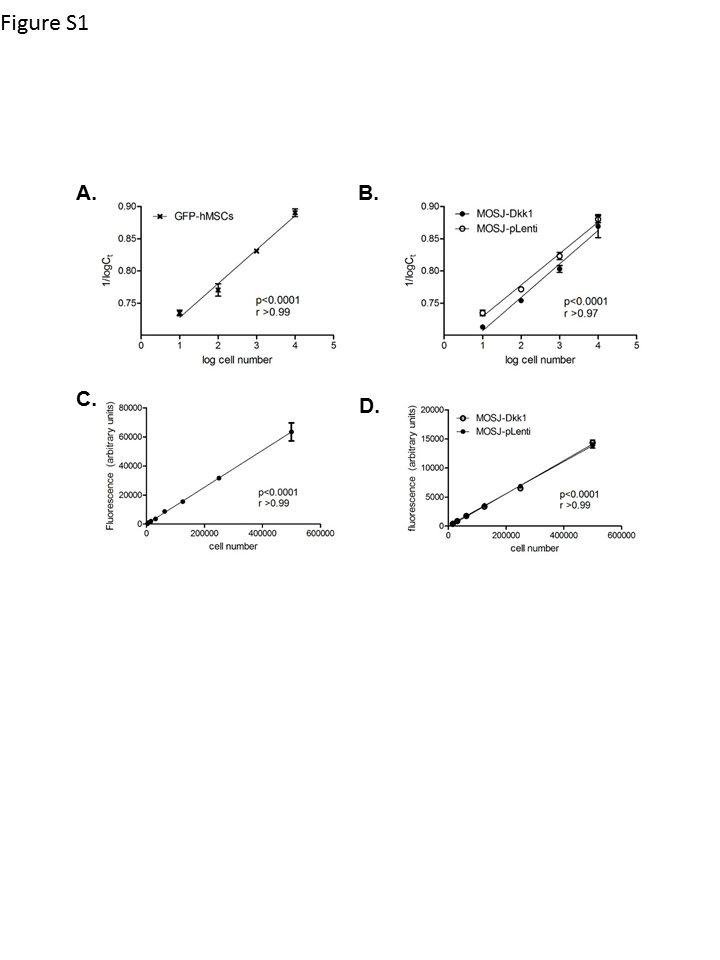


**Figure S3: Use of qRT-PCR and fluorescence readings to enumerate GFP-labeled OEhMSCs and RFP-labeled MOSJ cells.**  **Panels a, b:** Use of qRT-PCR for enumeration of OEhMSCs and MOSJ cells with species-specific primers for GAPDH (*Ct* refers to cycle threshold). **Panels c, d:** Use of fluorescence from GFP or RFP to enumerate OEhMSCs and MOSJ cells respectively. In all cases, cell standards were enumerated by hemacytometer before measurements were taken. PCR measurements were employed to count cells in RWV cultures and fluorescence for monolayer cultures. Absolute cell numbers for experimental cultures determined by the plots above. **Statistics:** n=3 with means and standard deviations. Regression analysis performed by Pearson’s correlation.


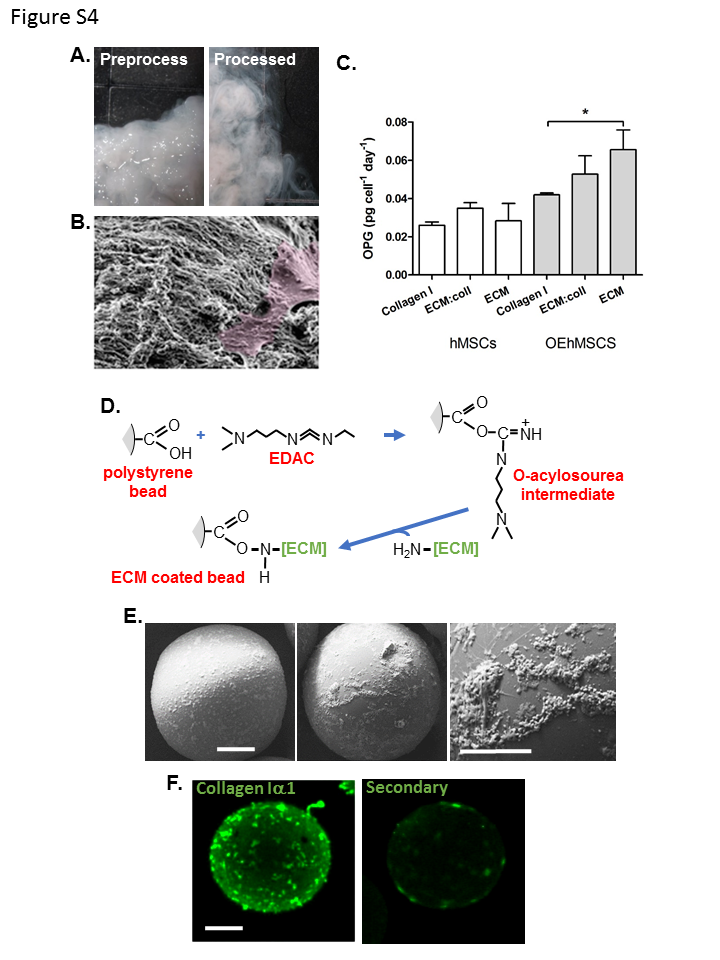


**Figure S4: Generation of ECM and ECM-coated beads. Panel A:** Appearance of recovered cell monolayers before (*Preprocess*) and after decellularization and processing (*Processed*). **Panel B:** Scanning electron micrograph of hMSC (*pink pseudocolor*) attached to processed ECM. **Panel C:** Normalized secretion of OPG from hMSCs and OEhMSCs attached to tissue culture plastic, rat tail collagen I, ECM, or a 1:1 mixture of collagen I and ECM (statistics: ANOVA with Tukey post test, *=p<0.05, n=4). **Panel D:** Reaction scheme employed to covalently attach ECM peptides to polystyrene beads. **Panel E:** *Left to right:* Electron micrograph of polystyrene beads that are uncoated (*bar* = 50 μm), coated with ECM, and coated with ECM at high power (*bar* = 20 μm). **Panel f:** Immunofluorescent staining for human type I collagen, a major constituent of the ECM. “*Secondary*” refers to a control that omits the primary antibody (*bar* = 75 μm).


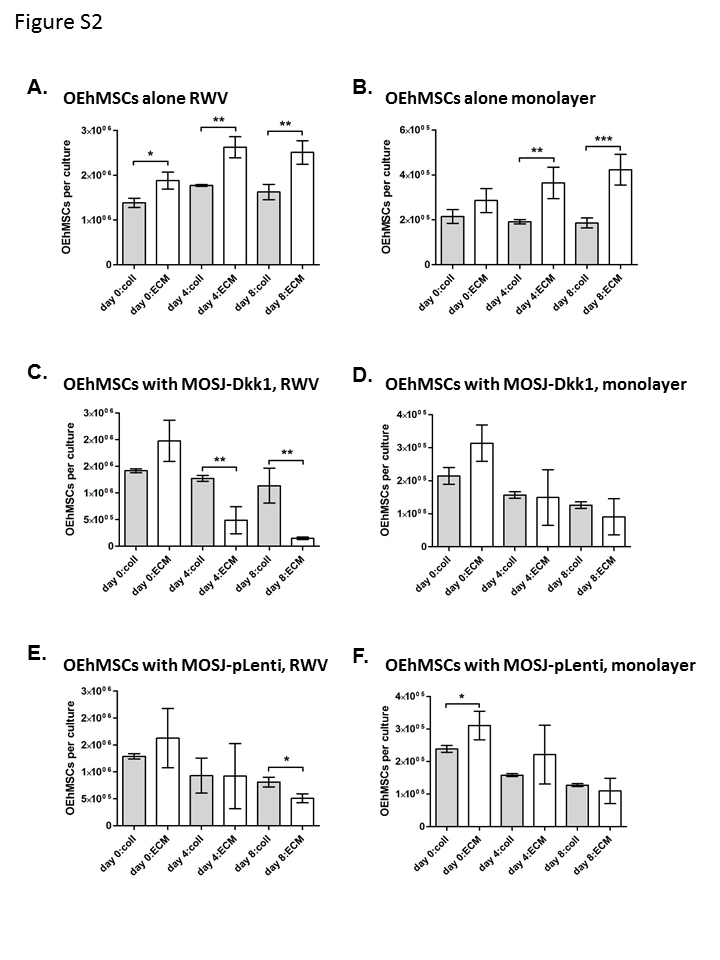


**Figure S5: OEhMSC recoveries after culture on OEhMSC derived ECM as compared to collagen I.**  **Panel a:** OEhMSC recoveries after culture in the RWV. **Panel b:** OEhMSC recoveries after monolayer culture. **Panel c:** OEhMSC recoveries after culture in the RWV with MOSJ-Dkk1 cells. **Panel d:** OEhMSC recoveries after monolayer culture with MOSJ-Dkk1 cells. **Panel e:** OEhMSC recoveries after culture in the RWV with MOSJ-pLenti cells. **Panel f:** OEhMSC recoveries after monolayer culture with MOSJ-pLenti cells. **Statistics:** n=3 for a,c and e, and n=4 with b, d and f. Plotted with means and standard deviations, statistical analysis ANOVA with Bonferroni selected pairs analysis, p<0.05 *, p<0.01 **, p<0.005 ***. All values refer to 50 cm^2^ growth area.


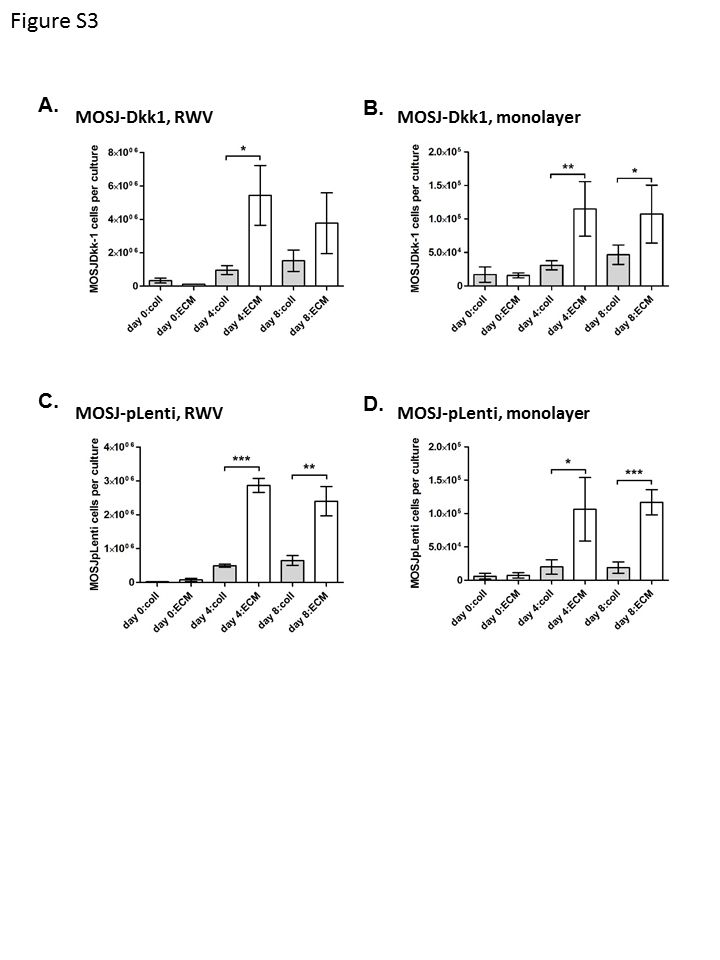


**Figure S6: MOSJ recoveries after culture on OEhMSC derived ECM as compared to collagen I.** **Panel a:** MOSJ-Dkk1 recoveries after culture in the RWV. **Panel b:** MOSJ-Dkk1 recoveries after monolayer culture. **Panel c:** MOSJ-pLenti recoveries after culture in the RWV. **Panel d:** MOSJ-pLenti recoveries after monolayer culture. **Statistics:** n=3 for a and c, and n=4 with b and d. Plotted with means and standard deviations, statistical analysis ANOVA with Bonferroni selected pairs analysis, p<0.05 *, p<0.01 **, p<0.005 ***. All values refer to 50 cm^2^ growth area.

**Supplementary Table 1: Primers and PCR conditions utilized in this study.**

| **TARGET** | **SEQUENCE** | **CONDITIONS** | **NOTES** |
| --- | --- | --- | --- |
| Human GAPDH | FOR ctctctgctcctcctgttcgac  REV tgagcgatgtggctcggct | SYBR-green 60^o^C | (2)  RTPrimerDB ID_1242* |
| Murine GAPDH | FOR catggccttccgtgttccta  REV gcggcacgtcagatcca | SYBR-green 60^o^C | RTPrimerDB ID_473* |
| Human collagen I | FOR gaacgcgtgtcatcccttgt  REV gaacgaggtagtctttcagcaaca | SYBR-green 60^o^C | RTPrimerDB ID_1089* |
| Human Runx2 | FOR gcaaggttcaacgatctgaga  REV tccccgaggtccatctactg | SYBR-green 60^o^C | (3) |
| Human Osx1 | FOR gtgggcagctagaagggagt  REV aattagggcagtcgcagga | SYBR-green 60^o^C | (3) |
| Human axin | FOR caggacactgctctctcagattca  REV tcacaacagcctttgcaggg | SYBR-green 60^o^C | (4) |
| Human BMP2 | FOR cccagcgtgaaaagagag  REV gagaccgcagtccgtcta | SYBR-green 50^o^C | Designed in this study |
| *Sourced from the RTPrimerDB database:  http://wwww.medgen.ugent.be/rtprimerb (5) | | | |

**Supplementary Table 2: Osteogenic genes upregulated by osteogenically enhanced hMSCs in response to ECM attachment.** ENTREZ refers to the Entrez gene ID accession number.

| **GENE NAME** | **ENTREZ** |
| --- | --- |
| Homo sapiens bone morphogenetic protein 2 (BMP2), mRNA. | 650 |
| periostin(POSTN) | 10631 |
| decorin(DCN) | 1634 |
| glycoprotein nmb(GPNMB) | 10457 |
| superoxide dismutase 2, mitochondrial(SOD2) | 6648 |
| RAS like proto-oncogene A(RALA) | 5898 |
| Ras related GTP binding C(RRAGC) | 64121 |
| SMG1, nonsense mediated mRNA decay associated PI3K related kinase(SMG1) | 23049 |
| Homo sapiens tumor necrosis factor receptor superfamily, member 11b (TNFRSF11B), mRNA. *Osteoprotegerin.* | 4982 |
| 2-aminoethanethiol dioxygenase(ADO) | 84890 |
| connective tissue growth factor(CTGF) | 1490 |
| phosphatidylinositol binding clathrin assembly protein(PICALM) | 8301 |
| LRR binding FLII interacting protein 1(LRRFIP1) | 9208 |
| transmembrane 9 superfamily member 3(TM9SF3) | 56889 |
| leucine rich repeat containing 58(LRRC58) | 116064 |
| Homo sapiens bone morphogenetic protein receptor, type II (serine/threonine kinase) (BMPR2), mRNA. | 659 |
| coiled-coil serine rich protein 2(CCSER2) | 54462 |
| N-acetylglucosamine-1-phosphate transferase alpha and beta subunits(GNPTAB) | 79158 |
| plastin 3(PLS3) | 5358 |
| heat shock protein family A (Hsp70) member 9(HSPA9) | 3313 |
| protein tyrosine phosphatase type IVA, member 1(PTP4A1) | 7803 |
| ribosomal L24 domain containing 1(RSL24D1) | 51187 |
| TSC22 domain family member 1(TSC22D1) | 8848 |
| fibronectin 1(FN1) | 2335 |
| lumican(LUM) | 4060 |
| ATPase phospholipid transporting 11B (putative)(ATP11B) | 23200 |

**Supplementary Table 3: Bone marrow genes upregulated by osteogenically enhanced hMSCs in response to MSC extracellular matrix attachment.**

| **GENE NAME** | **ENTREZ** |
| --- | --- |
| 2-aminoethanethiol (cysteamine) dioxygenase | 84890 |
| ATPase, class VI, type 11B | 23200 |
| COP9 constitutive photomorphogenic homolog subunit 8 (Arabidopsis) | 10920 |
| KIAA1128 | 54462 |
| N-acetylglucosamine-1-phosphate transferase, alpha and beta subunits | 79158 |
| Ras-related GTP binding C | 64121 |
| SMG1 homolog, phosphatidylinositol 3-kinase-related kinase (C. elegans) | 23049 |
| SMT3 suppressor of mif two 3 homolog 1 (S. cerevisiae); SUMO1 pseudogene 3 | 474338 |
| TSC22 domain family, member 1 | 8848 |
| adenosylhomocysteinase-like 1 | 10768 |
| asparaginyl-tRNA synthetase | 4677 |
| basic leucine zipper and W2 domains 1 pseudogene 1; basic leucine zipper and W2 domains 1 like 1; basic leucine zipper and W2 domains 1 | 151579 |
| calmodulin 3 (phosphorylase kinase, delta); calmodulin 2 (phosphorylase kinase, delta); calmodulin 1 (phosphorylase kinase, delta) | 805 |
| connective tissue growth factor | 1490 |
| cornichon homolog (Drosophila) | 10175 |
| cystatin C | 1471 |
| decorin | 1634 |
| fibronectin 1 | 2335 |
| gap junction protein, alpha 1, 43kDa | 2697 |
| glycoprotein (transmembrane) nmb | 10457 |
| heat shock 70kDa protein 9 (mortalin) | 3313 |
| isoleucyl-tRNA synthetase | 3376 |
| kinase D-interacting substrate, 220kDa | 57498 |
| leucine rich repeat (in FLII) interacting protein 1 | 9208 |
| leucine rich repeat containing 58 | 116064 |
| lumican | 4060 |
| matrin 3 | 9782 |
| periostin, osteoblast specific factor | 10631 |
| phosphatidylinositol binding clathrin assembly protein | 8301 |
| plastin 3 (T isoform) | 5358 |
| pleckstrin and Sec7 domain containing 3 | 23362 |
| praja ring finger 2 | 9867 |
| protein tyrosine phosphatase type IVA, member 1 | 7803 |
| protein tyrosine phosphatase, non-receptor type 12 | 5782 |
| ribosomal L24 domain containing 1; similar to ribosomal protein L24-like | 51187 |
| ribosomal protein S7; ribosomal protein S7 pseudogene 11; ribosomal protein S7 pseudogene 4; ribosomal protein S7 pseudogene 10 | 100128060 |
| similar to transmembrane protein 167A; transmembrane protein 167A | 100129118 |
| spastic paraplegia 21 (autosomal recessive, Mast syndrome) | 51324 |
| superoxide dismutase 2, mitochondrial | 6648 |
| tRNA nucleotidyl transferase, CCA-adding, 1 | 51095 |
| transmembrane 9 superfamily member 3 | 56889 |
| v-ral simian leukemia viral oncogene homolog A (ras related) | 5898 |

1. Dominici M*, et al.* (2006) Minimal criteria for defining multipotent mesenchymal stromal cells. The International Society for Cellular Therapy position statement. *Cytotherapy* 8(4):315-317.

2. Carraro G, Albertin G, Forneris M, & Nussdorfer GG (2005) Similar sequence-free amplification of human glyceraldehyde-3-phosphate dehydrogenase for real time RT-PCR applications. *Mol Cell Probes* 19(3):181-186.

3. Schaap-Oziemlak AM*, et al.* (2010) MicroRNA hsa-miR-135b regulates mineralization in osteogenic differentiation of human unrestricted somatic stem cells. *Stem Cells Dev* 19(6):877-885.

4. Bilir B, Kucuk O, & Moreno CS (2013) Wnt signaling blockage inhibits cell proliferation and migration, and induces apoptosis in triple-negative breast cancer cells. *Journal of translational medicine* 11:280.

5. Pattyn F, Speleman F, De Paepe A, & Vandesompele J (2003) RTPrimerDB: the real-time PCR primer and probe database. *Nucleic Acids Res* 31(1):122-123.
