# Supplementary figures and images for: Three-dimensional in vitro modeling of malignant bone disease recapitulates experimentally accessible mechanisms of osteoinhibition
Source: Cell Death Dis. 2018 Nov 26;9(12):1161. doi: 10.1038/s41419-018-1203-8 (PMC6255770; doi:10.1038/s41419-018-1203-8)

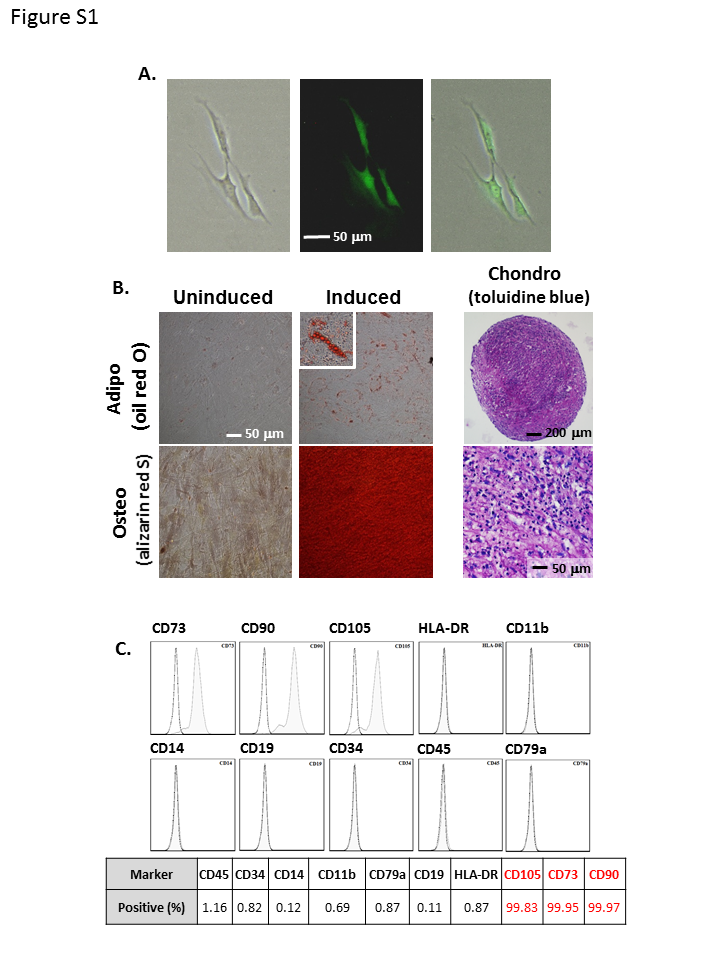

Supplement: Supplementary file 2 — Figure S1 [file 41419_2018_1203_MOESM2_ESM.tif]

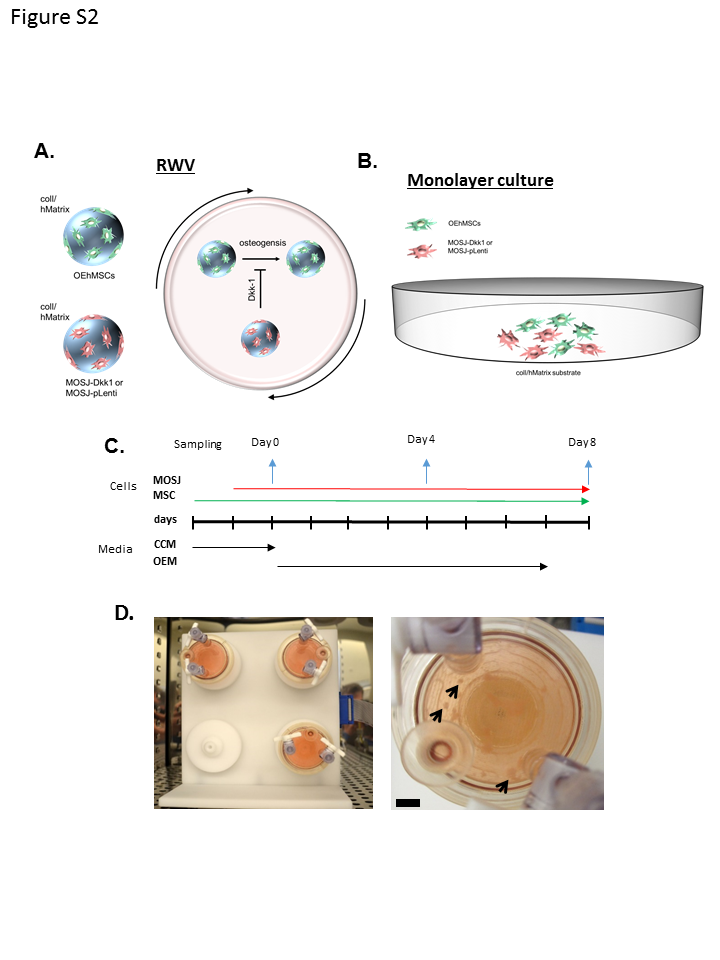

Supplement: Supplementary file 3 — Figure S2 [file 41419_2018_1203_MOESM3_ESM.tif]

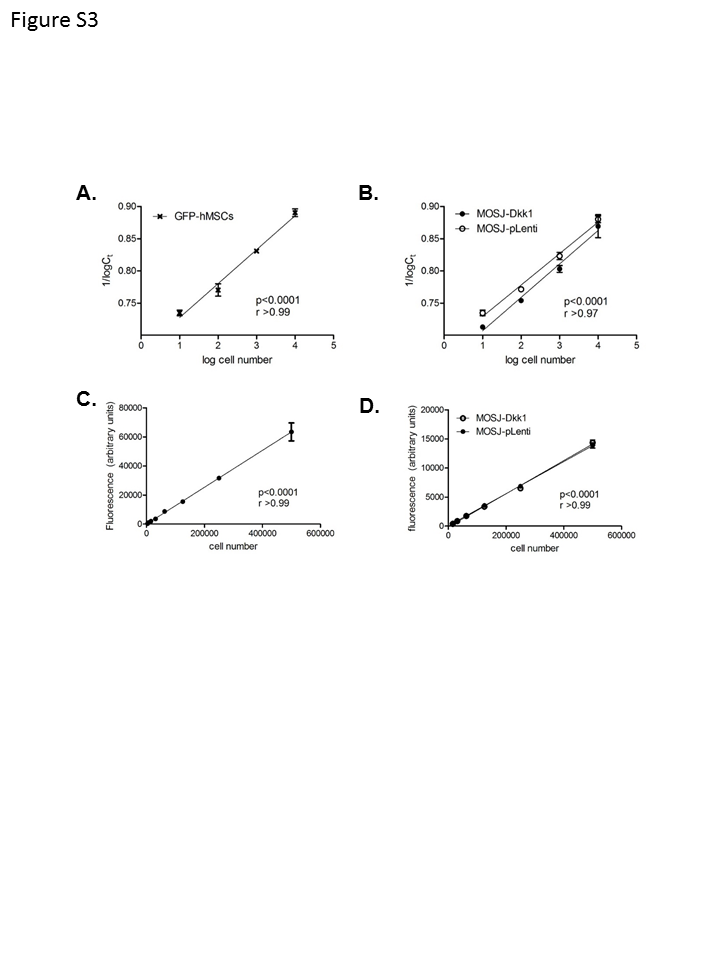

Supplement: Supplementary file 4 — Figure S3 [file 41419_2018_1203_MOESM4_ESM.tif]

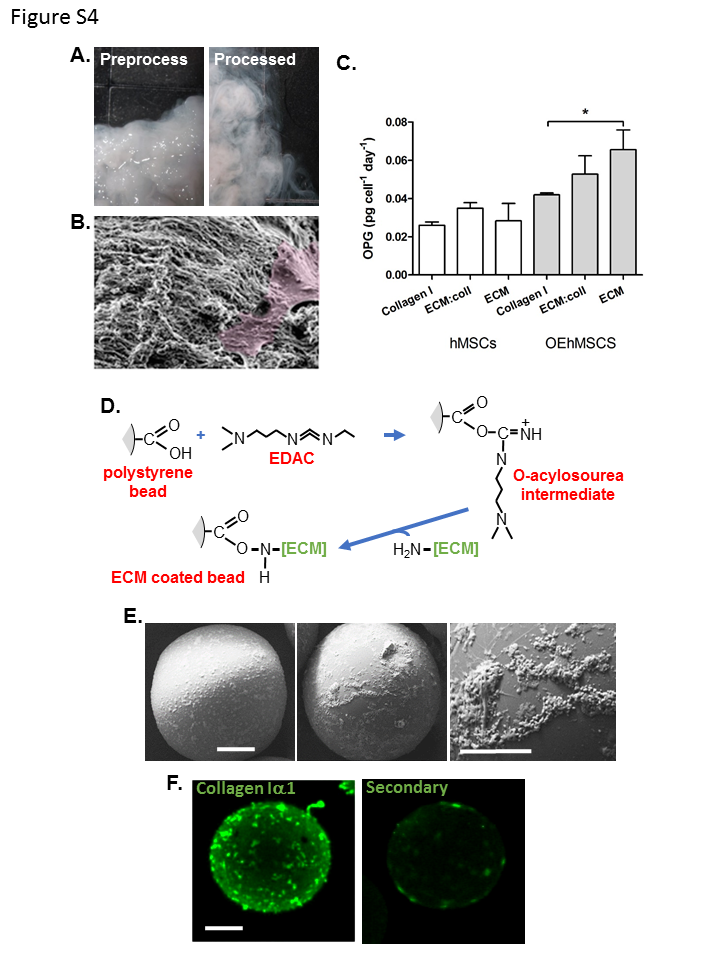

Supplement: Supplementary file 5 — Figure S4 [file 41419_2018_1203_MOESM5_ESM.tif]

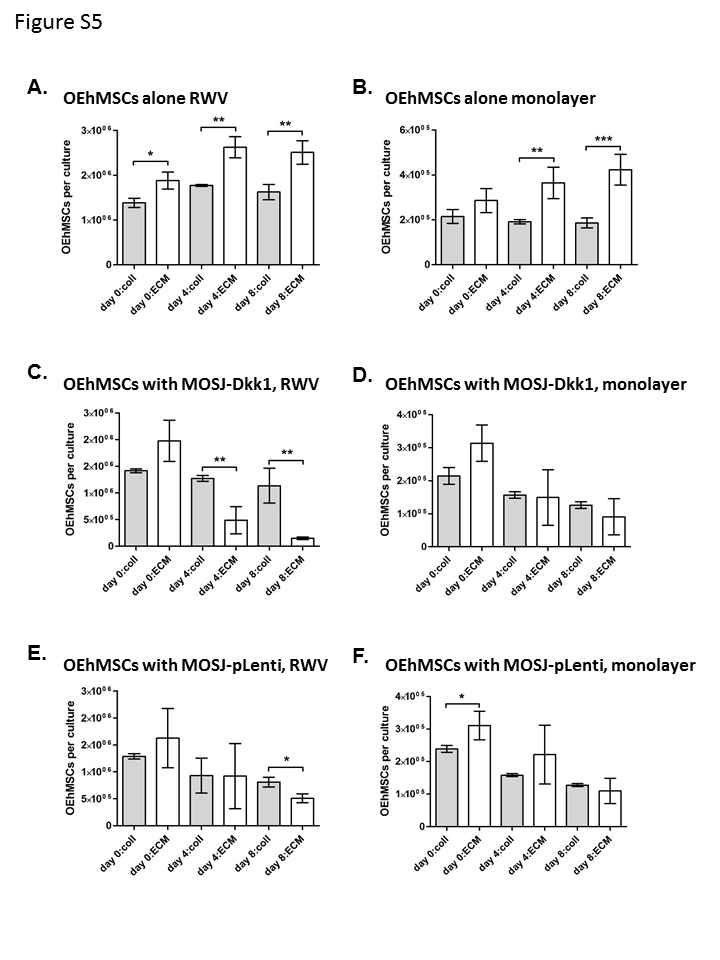

Supplement: Supplementary file 6 — Figure S5 [file 41419_2018_1203_MOESM6_ESM.tif]

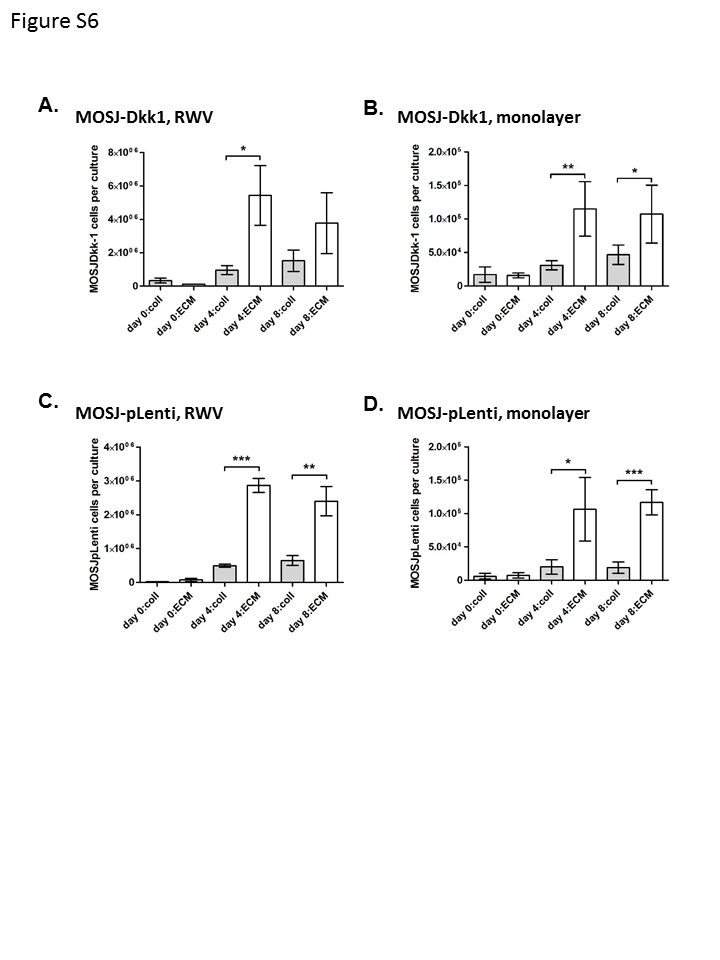

Supplement: Supplementary file 7 — Figure S6 [file 41419_2018_1203_MOESM7_ESM.tif]
